# Coextinctions dominate future vertebrate losses from climate and land use change

**DOI:** 10.1126/sciadv.abn4345

**Published:** 2022-12-16

**Authors:** Giovanni Strona, Corey J. A. Bradshaw

**Affiliations:** ^1^ European Commission, Joint Research Centre (JRC), Ispra, Italy.; ^2^ Faculty of Biological and Environmental Sciences, University of Helsinki, Viikinkaari 1, Biocentre 3, 00790 Helsinki, Finland.; ^3^ Global Ecology, College of Science and Engineering and ARC Centre of Excellence for Australian Biodiversity and Heritage, Flinders University, GPO Box 2100, Adelaide, South Australia 5001, Australia.

## Abstract

Although theory identifies coextinctions as a main driver of biodiversity loss, their role at the planetary scale has yet to be estimated. We subjected a global model of interconnected terrestrial vertebrate food webs to future (2020–2100) climate and land-use changes. We predict a 17.6% (± 0.16% SE) average reduction of local vertebrate diversity globally by 2100, with coextinctions increasing the effect of primary extinctions by 184.2% (± 10.9% SE) on average under an intermediate emissions scenario. Communities will lose up to a half of ecological interactions, thus reducing trophic complexity, network connectance, and community resilience. The model reveals that the extreme toll of global change for vertebrate diversity might be of secondary importance compared to the damages to ecological network structure.

## Teaser

Ecological dependencies amplify the effects of environmental degradation and promote vertebrate community collapse.

## INTRODUCTION

The planet has entered the sixth mass extinction (*1*–*5*). There are multiple causes underlying the rapid increase in observed and modeled extinction rates in recent times, of which land-use change, overharvesting, pollution, climate change, and biological invasions figure as dominant processes (*6*). However, assessing the relative importance and the realistic impact of such drivers at the global scale remains a challenge. Another aspect rendering assessment difficult are the synergies between drivers—a species might go extinct for multiple, simultaneous reasons, and in such contexts, ecological interactions play a fundamental role in predicting its fate (*7*). Growing recognition of the importance of species interactions in promoting the emergence of biodiversity in complex natural communities implies that an additional, fundamental component of biodiversity loss is represented by the amplification of primary extinctions across ecological networks. Coextinction—the loss of species caused by direct or indirect effects stemming from other extinctions—is now recognized as a major contributor to global biodiversity loss, strongly amplifying the effect of primary (e.g., climate-driven) extinctions (*8*–*11*).

Networks of ecological interactions are central to global patterns of diversity loss not only because coextinctions can be triggered by other extinction drivers, but also because network structure and dynamics might modulate several processes that can either reduce or increase extinction rate. For example, it is intuitive that a species’ success in colonizing a new area depends strongly on its ability to exploit local resources while simultaneously escaping enemies (predators and parasites). The addition of the new species might also initiate substantial changes to and have important cascading effects in the local network. Ignoring the structure of ecological networks and how they reconfigure as their constituent diversity changes therefore gives a possibly misleading view of the future of global diversity.

Previous attempts to predict the future of global diversity in the face of climate change and habitat modification have only considered the direct effects of these drivers on species (typically on single taxonomic groups), without explicitly accounting for ecological interactions. For instance, Thomas *et al.* (*12*) used projections of species’ distributions and species-area relationships to predict extinction rates for 20% of Earth’s surface, and Malcolm *et al.* (*13*) applied both species-area and endemic-area relationships to predictions of biome shift under climate change in Biodiversity Hotspots. van Vuuren *et al.* (*14*) also applied species-area relationships to vascular plants to project extinctions under different land-use and climate-change scenarios within the Millennium Ecosystem Assessment, and Jetz *et al.* (*15*) used a similar approach for birds. Others have applied analogous techniques to many other taxa, including lizards (*16*), crop wild relatives (*17*), chelonians (*18*), bird, amphibians, and corals (*19*). Later, Warren *et al.* (*20*) applied point-process and global circulation models to predict climate change–induced shifts in species’ distributions, and Urban (*21*) did a meta-analysis (including many of the studies cited above) to predict extinction rates of various taxa under several climate-change scenarios. Despite this extensive research foundation, future inferences of biodiversity’s fate over the coming century are likely to underestimate extinctions arising from global change (*11*).

Apart from the obvious modeling and computational challenges to incorporate interactions among species, the main reason why there are few studies accounting for interactions is that obtaining sufficient data in most communities is intractable. Therefore, global-scale modeling of entire ecosystems appears to be the only viable solution, even if a challenging one (*11*, *22*). Recent developments in network approaches have shown that potential ecological interactions can be derived by applying different techniques (e.g., machine learning) to available datasets on species distribution and ecology (*23*, *24*). In previous work (*11*), we built on that idea to generate global-scale models of biodiversity by including species interactions using virtual species constructed to follow real-world archetypes. In such synthetic approaches, a virtual species is a plausible ecological entity that has a combination of ecological traits consistent with real-world species despite not corresponding exactly to them.

There are several advantages in using virtual species in this manner. The first is that once the rules have been set to generate virtual species, current gaps and biases in biodiversity sampling cease to be a limitation; we can use virtual species to populate the entire Earth and generate plausible ecological communities, even in areas where data on local diversity are scarce or missing. Second, virtual species avoid preconceptions (and biases) about current biodiversity patterns, permitting instead a focus on the processes involved in change. Here, we can populate an entire virtual planet with species, let them develop communities based on a modest set of realistic ecological rules and assumptions, and then explore the emerging patterns. With such an approach, real-world data serve as a template for generating the virtual species and for identifying the basic ecological rules controlling community dynamics and as a benchmark with which to validate the realism of modeled predictions.

We previously demonstrated how coextinctions increase the pace of annihilation of life on Earth by up to 10 times relative to primary extinctions, but only in the face of catastrophic, no-return environmental change modeled as either extreme planetary heating or cooling (*11*). Although an instructive proof of concept, that model contained many simplifications and was applied to (hopefully) unrealistic scenarios of global change. Building on that original approach, here we developed a more complex, and ecologically realistic dynamic model to represent all terrestrial vertebrate communities with which we project future biodiversity trends. By accounting for both primary extinctions and their resulting coextinctions, the model predicts the cumulative toll on global biodiversity of different climate and land-use change projections up to 2100 at a spatial scale of 1° × 1° and at a monthly temporal resolution. In addition to providing estimates of potential global diversity loss, the model quantifies the relative contribution of the different extinction drivers at the global scale for the first time.

## RESULTS

**Fig. 1.**
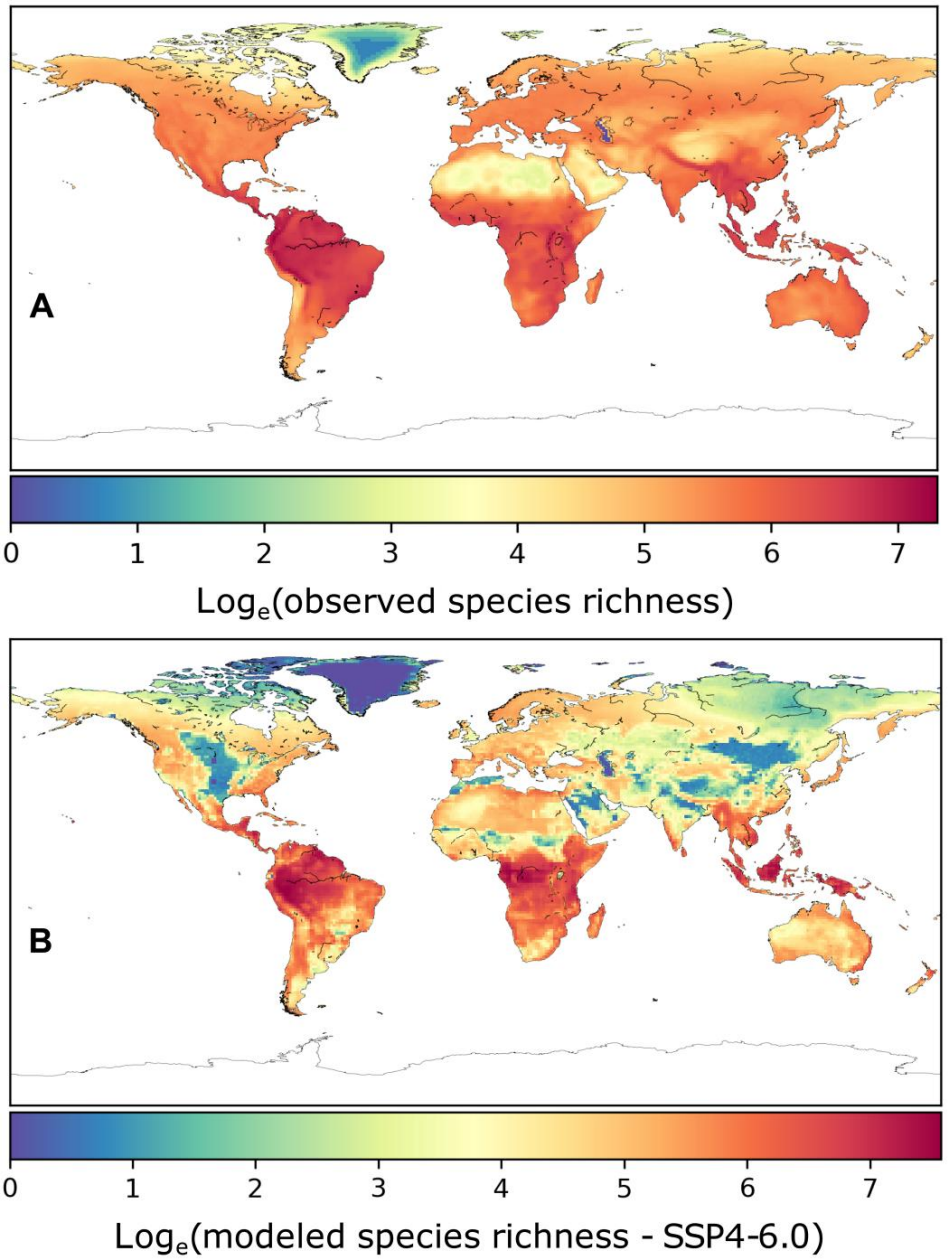
Observed versus modeled vertebrate diversity in 2020 in the CMIP6 SSP4-6.0 scenario. Observed diversity (**A**) is calculated by overlaying distributional ranges of all vertebrates used as an ecological mold for the virtual species (see Materials and Methods). Modeled diversity (**B**) corresponds to the log*_e_*-transformed number of virtual species per locality (averaged across 100 model replicates). The distribution of the virtual species does not result directly from the distribution of real-world vertebrates. Instead, the modeled diversity is an emergent property of the simulated system, stemming from the interaction between virtual species and the local virtual community/food web and environment. Map resolution is 1° × 1°. Maps for initial diversity in the other climate scenarios (CMIP6 SSP2-4.5 and SSP5-8.5), consistent with this one, are provided in figs. S2 and S3.

A limited set of assumptions regarding species’ ecology (niche + interactions) in the model generates a realistic map of initial (i.e., 2015) terrestrial vertebrate diversity that matches contemporary diversity patterns at the global scale (Fig. 1 and figs. S1 to S3; in all climate-projection scenarios, Pearson’s *r* > 0.6 and a slope ≈ 1 when regressing species richness in the model in each 1° × 1° cell in the global grid against a proxy for “true” diversity obtained by overlaying distributional ranges of 21,143 real-world vertebrate species; see Materials and Methods for details). The consistency in observed versus simulated global diversity patterns provides support for the ecological realism of our network model, showing that it can reproduce plausible, broad-scale patterns of diversity. Notably, our modeled diversity map shows a finer-grained variation in diversity patterns derived from the interplay of climate, land use, and ecological interactions compared to maps of range overlap. The model also produced realistically structured networks and a plausible regional distribution of species body masses (figs. S4 and S5).

**Fig. 2.**
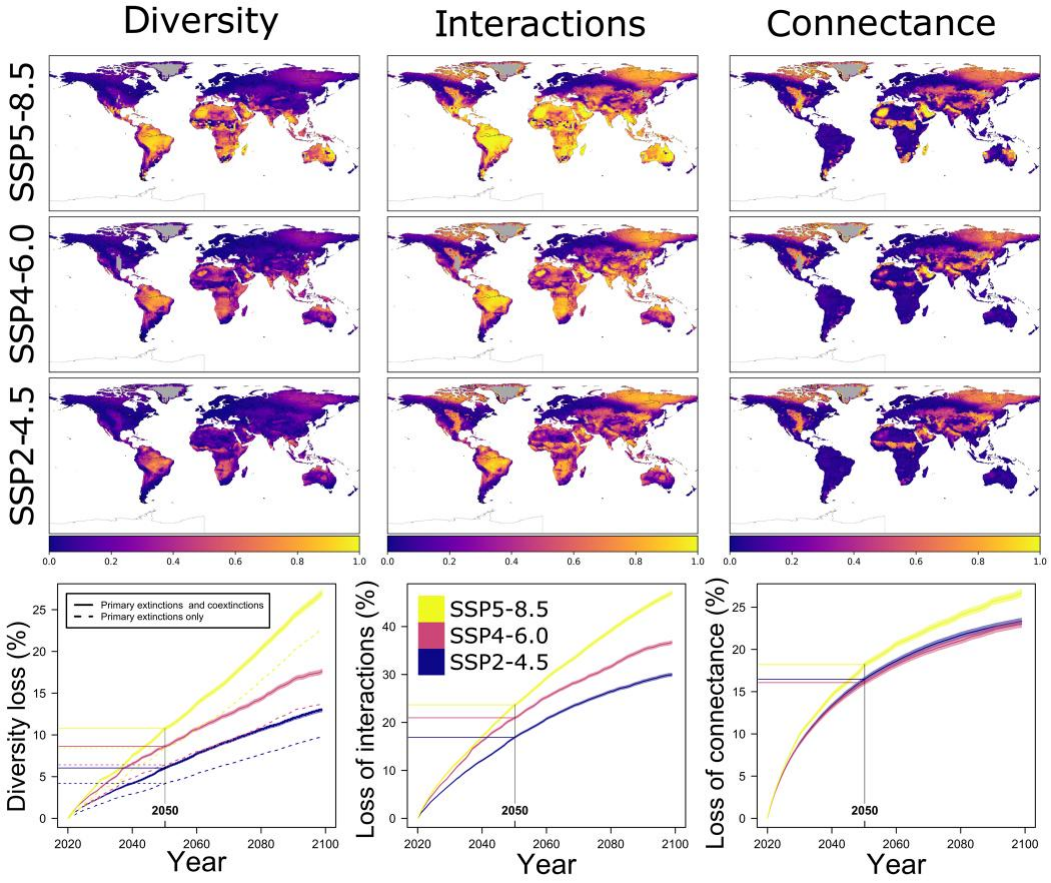
Simulated, relative local loss of terrestrial vertebrate diversity, interactions, and connectance by 2100 compared to 2020 under different climatic projections. Maps show local loss in 2100 relative to initial conditions, while plots show the average loss across all Earth localities per year from 2020 to 2100. In both maps and plots, results are the average of 100 replicates (for the plots, first, we averaged local loss in the 100 replicates and then averaged local loss values across all localities. Shaded areas are 95% confidence intervals, showing the variation across localities in the average map obtained with 100 simulations).

Our model predicted global biodiversity to experience local losses by 2050 ranging [across different CMIP6 carbon-emissions scenarios (*25*)] from 6.0% (± SE = 0.1%, SSP2-4.5) to 10.8% (± 0.1%, SSP5-8.5) on average compared to initial diversity (and from 13.0 ± 0.1% to 27.0 ± 0.2% by 2100; Fig. 2, left column). In all climate scenarios, climate change was directly responsible (see also Discussion) for the most substantial fraction of local extinction events (62.1 ± 7.2% SD in the intermediate SSP4-6.0 scenario), followed by secondary extinctions (20.3 ± 5.4%), local extirpations due to overcompetition by colonizers (13.9 ± 4.1%), and land-use change (3.7 ± 3.1%; see fig. S8 and table S1).

Tracking network structure as it varied through time, we could also quantify how the loss of species resulted in the loss of network interactions (edges). This loss of edges was substantial, averaging 23.6 ± 0.2% by 2050 in the worst-case scenario (47.0 ± 0.3% by 2100; Fig. 2, middle column). The high rate of loss of network edges resulted in an overall strong reduction in network connectance (computed as number of edges ÷ squared number of nodes, because we are not preventing any interaction from occurring a priori, including cannibalistic interactions). Specifically, connectance declined 18.2 ± 0.2% by 2050 on average in the worst-case climate-change scenario (26.7 ± 0.2% by 2100; Fig. 2, right column). This result is consistent with the expectation that the vulnerability of species to climate change will be insensitive to their position and role in local networks, making the observed trajectories of network disassembly worse than those expected under stable conditions (see fig. S9).

Networks also became “shorter” in diameter (the longest of all the shortest paths connecting any two nodes in a network), with a reduction of 11.7 ± 0.1% by 2050 and 26.0 ± 0.2% by 2100 in the worst-case scenario (fig. S10A), and increasingly fragmented, with a loss of 12.8 ± 0.1% by 2050 (23.5 ± 0.1% by 2100) of the fraction of nodes in the largest, weakly connected component of the network (i.e., the largest cluster of nodes having at least one path to one of the nodes in the same cluster) in the worst-case climate-change scenario (fig. S10B). In terms of community structure and composition, we observed a moderate reduction in the average and maximum trophic level in local communities (12.0 ± 0.1% and 13.2 ± 0.1%, respectively, by 2100 in the worst-case climate-change scenario), and a large reduction in the average and maximum body mass of local (vertebrate) species (26.0 ± 0.2% and 38.3 ± 0.2%, respectively; fig. S8). As expected, larger species in high trophic levels will be the most threatened by future climate and land use changes. The reduction in body size and trophic level will be mainly driven by coextinction processes, with weaker patterns in the control simulations where we modeled primary extinctions only (i.e., those occurring due to climate and land-use change, and biological invasions), but not their cascading effects through trophic links (fig. S11).

**Fig. 3.**
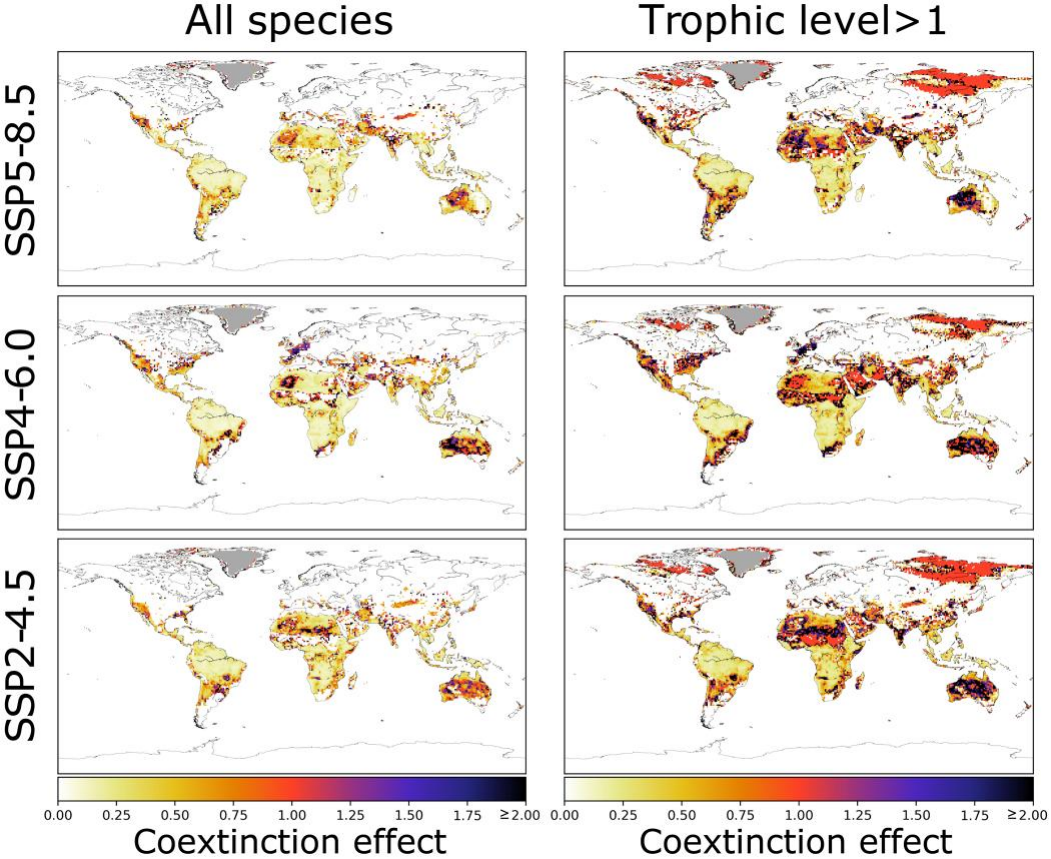
Coextinction effect by 2050. This is quantified as the percentage increase in diversity loss in the coextinction scenario compared to the reference (primary extinctions only) scenario. To ease visualization and comparisons, we set the maximum value of the color bar to 2 (i.e., 200% increase), but black pixels indicate all values ≥ 200%.

When we compared the simulations including coextinction events to the controls that only accounted for primary extinctions, the average effects of coextinctions (measured as the percentage decrease in biodiversity between the coextinction and control simulations) were 27.5 ± 1.5%, 39.2 ± 2.5%, and 21.8 ± 0.6% by 2050 (27.1 ± 2.0%, 34.0 ± 4.0%, and 18.1 ± 0.7% by 2100) in the three climate-change scenarios SSP2-4.5, SSP4-6.0, and SSP5-8.5, respectively (Fig. 3, left column). However, a potentially overoptimistic assumption of the model is that herbivores and invertebrate feeders never run out of plant and insect biomass. Our model treats insects and plants as nondepletable resources, despite growing evidence for invertebrate declines globally (*26*). This implies that both consumers capable of only using vertebrates and those also using invertebrates (insectivores and omnivores) are “invulnerable” to bottom-up coextinctions—extinctions of consumers triggered by resource depletion, which is the most common expectation for the mechanism underlying secondary extinctions (*8*–*11*)—and could therefore go extinct “only” due to climate and land-use change, as well as top-down network effects and outcompetition by new colonizers.

Focusing instead on “vulnerable” species only—those at a trophic level higher than strict herbivores and insectivores—to avoid potentially underestimating the effect of coextinctions, diversity loss and the differences between the coextinction and control scenarios rise substantially (Fig. 3, right column). Here, the effect of coextinctions amplified biodiversity loss relative to the primary-extinction scenario by 115.3 ± 3.9%, 148.0 ± 6.3%, and 107.8 ± 4.3% by 2050 (173.7 ± 9.4%, 184.2 ± 10.9%, and 144.4 ± 6.1% by 2100) in the three climate-change scenarios, with 90^th^ percentiles of 191.2, 223.7, and 172.4% by 2050 (250.0, 237.3, and 223.2% by 2100), respectively. In all climate change scenarios (and in both the simulations accounting for coextinctions and those not), the average rate of diversity loss was higher from 2020 to 2050 than from 2051 to 2100 (Fig. 2, bottom left panel, and fig. S12). Consequently, the greatest changes in diversity and network structure are already appreciable before 2050 (fig. S13), highlighting how the bleakest time for natural communities might be imminent and that the next few decades will be decisive for the future of global biodiversity.

Intriguingly, the strongest coextinction effect is predicted in the intermediate-severity SSP4-6.0 scenario. These relatively lower predicted losses in the worst-case SSP5-8.5 climate change projection are explained by the greater number of species in that scenario that are wiped out by primary extinctions arising directly from intolerance to climate change compared to milder climate-change projections, a characteristic that slightly reduces the relative importance of coextinctions therein (Fig. 3, right column).

**Fig. 4.**
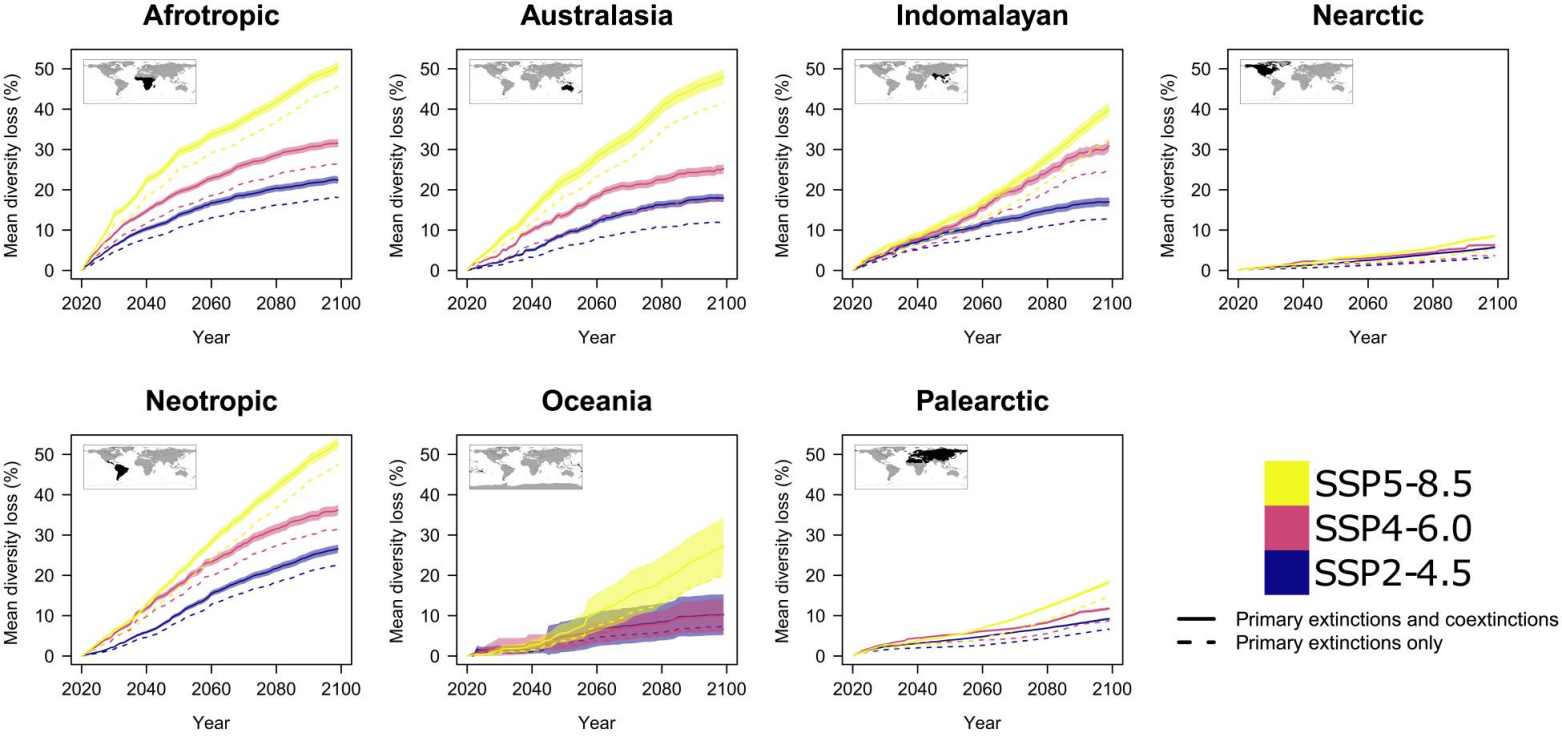
Diversity loss predicted to be higher in highly diverse biomes and lower in the Palearctic and Nearctic. Diversity loss is the % loss in species number per locality compared to 2020. We first averaged local loss in 100 replicates and then averaged local loss values across all localities in a given region. Solid and dashed lines are average values, while shaded areas indicate 95% confidence intervals, showing the variation across localities within a region in the average map obtained with 100 simulations. Equivalent maps for loss of interactions and connectance are provided in the Supplementary Materials.

When we examine network diversity and structure among biomes, most of the expected losses are predicted to occur in areas with the highest species richness. This shows that Biodiversity Hotspots not only have more species under threat and hence will lose more species (*27*, *28*) but that they will also experience the highest rates of loss due to coextinctions over the coming decades (Fig. 4). In general, and regardless of biogeographical region, diversity loss tended to increase with initial diversity (fig. S14). Consistent patterns were also predicted in the decline of network interactions (fig. S15) and connectance (fig. S16).

## DISCUSSION

Our virtual-Earth model reveals the magnitude of and mechanisms driving biodiversity loss expected from climate change and land conversion this coming century. These results not only suggest a much greater loss than previously anticipated (*21*), they also demonstrate that biodiversity loss will be accompanied by an additional weakening of community resilience via erosion of the connectance of ecological networks.

An important caveat is that while our virtual species are functionally realistic, they do not have taxonomic or phylogenetic meaning. Hence, our results reveal local changes in species diversity but do not provide information on global species extinctions per se. Neither does the model claim to produce an Earth replica, but instead aims to build an ecologically plausible Earth. Hence, the model cannot forecast Earth’s future but instead projects relative potential scenarios based on different assumptions (mainly carbon emissions) and reveals the underlying processes leading to those outcomes.

Examining the different drivers of extinction, our model reveals that the effect of climate change at the global scale is dominant, while land-use change played a comparatively minor role (fig. S8). However, in no way does that result refute the conclusion that land-use change is a major element of biodiversity loss; rather, it emphasizes that climate change is becoming more important. This emerges from two aspects of the model. First, we only considered relative land-use change from 2020 onward, meaning that the results reflect the relative future impact of land-use change, and not its dominant historical impact on biodiversity loss (*3*). Second, a strength of our model is that it can map extinctions everywhere on Earth. Even considering the extent of current human impacts, human presence and land-use change still directly affect only a small fraction of the total land inhabited by species. The area of primary and secondary cumulative land projected to be lost from 2020 to the end of the century for the worst-case climate change scenario (SSP5-8.5) is 8,000,000 km^2^ in total; however, this value represents only 6.5% of the global area in the simulation inhabited by at least one species in 2020 (~ 130,000,000 km^2^) (fig. S17).

It is also important to consider that the climatic projections take into account land-use change as one of the inputs to derive climatic variables. Therefore, the contribution of climate change to diversity loss also includes, in principle, the indirect effect of land-use change on climate. Our model does take this into account, as well as other potential indirect, detrimental effects of land-use change, such as increasing fragmentation and then possible alterations to species dispersal and colonization patterns. However, our analysis of the relative importance of extinction drivers focuses exclusively on the direct effects of climate change, land-use change, coextinctions, and biological invasions. Under these caveats, the direct contribution of habitat loss to global extinction is still high, albeit relatively lower than what climate change will occasion.

These results are also robust to different assumptions regarding the local impact of land-use change on diversity; these different choices do not change our overall conclusions (figs. S6, S7, and S18). The main results presented here refer to a model where we assumed a linear relationship between the fraction of primary and secondary land lost at each step in a given locality and the resulting loss of local diversity. More pessimistic scenarios assuming high diversity loss following moderate changes in land use led to more extinctions globally (with a threshold effect observed for *S*_LUC_ > 0.2; see Materials and Methods and fig. S27), but did not change our conclusions on the relative disproportionate importance of climate change.

By modeling food webs explicitly, our approach reveals synergies among extinction drivers (*7*), thereby confirming that overexploitation of resources by novel colonizers combined with climate change (i.e., biological invasions) will become a major cause of diversity loss worldwide (*29*, *30*). Our model therefore provides the first global quantitative assessment of the impacts of biological invasions on planetary diversity over the coming century.

An interesting outcome from the sensitivity analyses is that increasing the frequency and intensity of acclimation events in local populations can counterintuitively lead to a higher global extinction rate. The “adaptation” mechanism implemented in our model (see Materials and Methods) assumes that species can shift their niches to match the climate of the preceding year. Such a mechanism—although reducing the risk of extinction for species capable of adapting under a stable climatic regime—does not necessarily ensure protection against future changes. When we assume a high probability of adaptation (e.g., 0.5% of species shift their niche in all localities every year), the net effect is a reduction in average persistence of global diversity. This suggests that a strong and widespread adaptation to local conditions might both increase robustness toward steady conditions while simultaneously increasing vulnerability to change. This outcome is consistent with predictions based on a completely different approach (artificial life evolution simulations) showing that as ecological networks become more resilient to stable environmental conditions, they also become increasingly susceptible to change (*31*).

Our results confirm that coextinctions are fundamental drivers of mass extinctions (*5*–*11*) and suggest that previous large extinction events revealed from the fossil record would likely have been exacerbated beyond their primary environmental drivers via the negative feedbacks arising from ecological dependencies. Unless conservation practitioners rapidly start to incorporate the complexity of ecological interactions and their role in extinction processes in their planning, averting the ongoing biodiversity crisis will become an unachievable target.

## MATERIALS AND METHODS

### Model synopsis

Our simulated world consists of a data-driven system of interconnected terrestrial communities composed of virtual, yet ecologically plausible, vertebrate species whose ecology and functional traits obey rules that we derived from combining several large, real-world datasets. The virtual vertebrates in each locality are organized into food webs according to their trophic ecology, diet breadth, and functional traits, with links assigned on the basis of trophic rules derived from empirical data. This generates a dynamic, global-scale system where the patterns and process of food webs are an emerging property of ecological mechanisms inferred from empirical data. Our implementation of the networks’ responses to changes in species composition permits modeling and tracking communities at high spatial and temporal resolution, without the prohibitive challenge of modeling multispecies population dynamics and ecological interactions in thousands of sites hosting hundreds of species.

We tracked the future development of the virtual life planet from 2020 to 2100 at a spatial resolution of 1° × 1° latitude following three climate-projection scenarios [Coupled Model Intercomparison Project Phase 6 (CMIP6): Shared Socioeconomic Pathway (SSP) 2-4.5, SSP4-6.0, and SSP5-8.5] (*25*), running 100 replicates for each future climate-projection scenario. The model runs at a temporal increment of 1 month for climate and 1 year for spatially explicit land-use changes and food-web dynamics. We allowed species to disperse among localities, with the probability of colonization success depending on the geographic distance between the source and the target locality, compatibility between species’ tolerance limits and the environmental conditions (species-specific suitability) of the target locality, and the ability of a species to enter the local food web at the target site. Last, the model includes a function allowing for some species-specific adaptation to changing climate conditions (see Materials and Methods for all modeling details).

During the simulations, species went extinct owing to direct effects of environmental change (i.e., when no longer capable of tolerating local climatic conditions or following loss of primary land) or indirect (either bottom-up or top-down) effects propagating through network links. When a species went extinct, we explicitly accounted for the cascading effect of that extinction on its corresponding local food web. That is, interaction networks in the model continuously adapt and respond to changes in community composition. As species go extinct, and new species enter a local food web from different localities, nodes and links are added and removed, and interaction weights are recalculated on the basis of the new conditions.

Recalculating weights permits simulating ecological mechanisms fundamental for food web functioning, such as changes in a consumer’s diet and realized specialization driven by resource availability. The initial weights of a network are rescaled depending on resource availability and on the diversity of consumers having access to the different resources. Whenever something changes in the network (e.g., species are added or removed), weights are adjusted to reflect the new ecological setting. Thus, a consumer that was initially consuming a particular resource might switch a substantial proportion of interaction weight to a novel (more compatible) resource (note that such change is reversible—the consumer can reallocate interaction weights to the initial resource should the situation return to a previous state). Similarly, the diet breadth (in terms of the distribution of interaction weights from a consumer to the set of available resources) might change substantially when species are either lost or added to the network. This might involve one species initially preying mostly on vertebrates to switch its interactions to invertebrates only. In this way, our model can simulate species’ plasticity in response to altered conditions, with the coextinction risk of species changing as communities reorganize themselves in response to environmental and ecological change. The same set of rules permits simulating both positive and negative effects of colonizers, either rescuing local populations or increasing local diversity (see the Supplementary Materials), or driving local species to extinction due to overexploitation by the invader.

As a control in each simulation to assess the net effect of coextinctions on global diversity loss, we also ran a parallel experiment where we did not model coextinctions and the other network-related events, while maintaining all other ecological processes (primary extinctions, dispersal, and adaptation; also see the discussion in the Supplementary Materials). Further, model outputs were robust to assumptions of adaptive capacity (Supplementary Materials, figs. S6 and S7).

### Species climatic niche

We collected all available vertebrate ranges from the International Union for the Conservation of Nature (*32*) and BirdLife International (*33*). We rasterized the ranges on a 1° × 1° global grid and then retained only the species occurring in > 5 cells (terrestrial mammals: 4452; reptiles: 3903; amphibians: 3040; birds: 9748; total = 21,143). We referred to the CMIP6 global circulation model for temperature and precipitation predictions, using three different scenarios: SSP2-4.5, SSP4-6.0, and SSP5-8.5 (approximately equivalent to updated versions of CMIP5 scenarios RCP4.5, RCP6.0, and RCP8.5, respectively) (*25*). In particular, we used the multimodel ensembles provided by the Canadian Centre for Climate Modelling and Analysis (*34*), at a spatial resolution of 1° × 1° latitude/longitude degrees and at a monthly temporal resolution.

We modeled species’ niches independently in each climatic scenario by identifying all temperature and precipitation values in the target scenario intersecting a species’ range at a monthly interval from 2015 to 2019. We defined suitability (*p*) of a species to the target climatic condition *x* (a temperature or precipitation value) as

$$p=\left\{ \begin{array}{c} 1-\frac{1}{1+e^{-c(x-d)}},\mathrm{if}\;x\leq \bar{x}\\ \frac{1}{1+e^{-a(x-b)}},\mathrm{otherwise} \end{array}\right.$$

where $\bar{x}$ is the average of the monthly measures of the target climatic condition across all 1° × 1° localities within the species’ range in the period January 2015 to December 2019. For each species, we estimated the *a*, *b*, *c*, and *d* parameters so that

$$v=v_{\min}\vee v=v_{\max}\to p=p_{\mathrm{thresh}}=0.95$$

where *v*_min_ and *v*_max_ are the maximum and minimum recorded values, respectively, of the target climatic condition in the species’ range within the reference time interval, and *p*_thresh_ is an arbitrarily selected probability of species survival at the edges of the species tolerance limits. Here, we conservatively chose *p*_thresh_ = 0.95 assuming that a given species would have this probability of surviving when the local temperature equals either *v*_min_ or *v*_max._ To derive the *a*, *b*, *c*, and *d* parameters, we first set *a* = 0.0001 and $b=\frac{\log\left(\frac{p_{\mathrm{thresh}}}{1-p_{\mathrm{thresh}}}\right)+{av}_{\max}}{a}$.

We then incremented *a* progressively by 0.01 per step until $\frac{1}{1+e^{-a(x-b)}}$ ≤ 0.001. Similarly, we set *c* = 0.0001 and $d=\frac{\log\left(\frac{1-p_{\mathrm{thresh}}}{p_{\mathrm{thresh}}}\right)+{cv}_{\min}}{c}$, and we then incremented *c* progressively by 0.01 per step until $\frac{1}{1+e^{-c(x-d)}}$ ≥ 0.9998. This procedure generates an asymmetric, bell-shaped probability curve that has values close to 0 when conditions are optimal, a probability of extinction = 1 − *p*_thresh_ = 0.05 when the species is at its tolerance limits, and then it declines rapidly as the local variable of interest departs from the species’ tolerance. The two curves obtained for temperature and precipitation define a bidimensional niche where at any given set of climate values, the probability of survival is given by the minimum probability of survival for both variables (fig. S19).

This approach can be considered as an advanced take on the idea of bioclimatic envelopes (*35*) because it produces a progressive probability of local extinction as temperature and precipitation approach the most extreme conditions experienced in the species’ range. During model development, we compared the probability of extinction from our niche model with those from other methods using nonlinear approaches to identify suitability. Such tests showed that our approach produced a more plausibly progressive increment in extinction risk as local environmental conditions approach the hypothetical tolerance limits compared to the niches produced by other techniques. For instance, we compared the extinction risks produced by either our approach or a *RandomForest* regressor on a random sample of 100 vertebrate species across a continuous range of temperature and precipitation values (for a total of 400 unique combinations of temperature and precipitation covering all the ideal space from the minimum to the maximum values recorded on Earth in the reference period 2015–2019). Although we found overall consistency between the risk estimates yielded by the two approaches (Spearman’s ρ = 0.41; *P* << 2.2 × 10^−16^), *RandomForest* produced an unrealistic representation of the bidimensional niche space with abrupt jumps from low to high extinction probability (such as classical bioclimatic envelopes) (fig. S20).

Although related to concepts typical of ecological niche models that aim to predict species distributions from environmental/climatic variables, our approach does not suffer from their common limitations. Our derived niches model the risk of local disappearance based on temperature and precipitation, and not to predict species distribution. While subtle, the distinction makes our model robust to the most common weakness associated with ecological niche models (*36*). A major limitation is that climatic suitability is just one of the many factors determining species presence, yet correlative niche models often ignore biogeographic history, the presence of other species, and stochastic events. However, while a species might not occur in a perfectly suitable area, it almost certainly does not occur/persist in unsuitable locations. In contrast, our modeled species occurrences are the combined and explicit result of niche, dispersal, and interactions with other species—i.e., our model is not affected by the potential biases that might be derived from equating suitability with presence. Instead, our model applies the robust and logical assumption that species cannot persist in an unsuitable area to determine the risk of local extinction.

Our definition of local extinction risk in this context has a community-level interpretation: Niche predictions do not identify the most extreme conditions tolerable before physiological failure; rather, they are the likelihood that a local population will decline and eventually disappear when exposed to unfavorable conditions. This logic precluded deriving thermal tolerances directly from databases such as *GlobTherm* (*37*), as we did previously (*11*), because empirically derived limits probably overestimate the true population-level responses implicit in our model. However, the distribution of thermal tolerances we generated is consistent with that from *GlobTherm* (fig. S21), even if slightly shifted toward lower temperatures (a desirable attribute).

### Species trophic level and body-mass relationship

We obtained body mass for 31,098 species combining information from different sources (*38*–*41*). For a subset of those species, we also obtained data on trophic interactions by extracting all available resource-consumer interaction data from the *GloBI* online database (*42*) (714,465 at the time of the analysis retrieved from globalbioticinteractions.org/data). For a given species, we used the interaction data to identify both the maximum trophic level (measured as the maximum distance from a basal resource) and its trophic breadth (variation in trophic level of the consumed resources). We combined this information to generate two datasets: The first included body mass and environmental niche (temperature/precipitation tolerance) for 17,238 species; the second included trophic level, trophic breadth, and body mass for 1449 species. The second dataset gives a distribution of trophic level per body-size interval for each taxonomic group (fig. S22), which assigns trophic level to a given species according to its body size and major phylogenetic grouping by sampling randomly from the distribution of trophic level observed in similar-sized species within the same taxon (described in the following section).

### Creating virtual species

To incorporate as much plausible variation as possible in this stochastic model, we generated an independent set of virtual species at the beginning of each global-extinction simulation according to the following procedure. We extracted species from the dataset associating species’ niches to the respective body mass, with the sampling structured to match known relative vertebrate diversity according to the IUCN (*32*) (i.e., 5513 mammals, 7302 amphibians, 10,425 birds, and 10,038 reptiles). We then compared the body size of the selected species with all body sizes from the body-size and trophic datasets (in random order) until we found a species belonging to the same taxonomic grouping and with “matching” body size (see below), and then we associated the trophic information to the target species. In this way, we accounted for potential relationships between ecological niche, body mass, trophic level, and taxonomic group.

We considered that two body sizes were a match if

$$\frac{\max[m_{1},m_{2}]-\min[m_{1},m_{2}]}{\max[m_{1},m_{2}]}<r_{\mathrm{thresh}}$$

where *m*_1_ and *m*_2_ are the body size of the two species, and *r*_thresh_ = 0.01, a threshold for each species. If no match was found with this threshold, we progressively increased the threshold at steps of 0.01 until a match emerged. The average of final thresholds within a simulation was 0.065 ± 0.11 with a maximum of 0.96. The largest thresholds are associated with the smallest species, so that the average absolute difference in body mass between matched species is < 300 g.

We then sampled trophic level and breadth corresponding to body-size class and taxonomic group. From this point, the taxonomic identity of a species is no longer maintained in the simulations. We added functional traits to the species following Strona and Bradshaw (*11*) in the form of a random string of letters (sampled from the standard, 26-letter English alphabet) of size varying stochastically between 1 and 10, ideally corresponding to the virtual species phenotype.

At the beginning of each simulation, we created a 26 × 26 adjacency matrix filled with real numbers varying randomly between the limits of −1 and 1, indicating the compatibility between any two functional traits (alphabet letters) when considered in isolation. We quantified the compatibility between any two phenotypes by summing all the compatibility values obtained by all pairwise combinations of letters in each phenotype. We then normalized these values between 0 and 1 (by estimating the minimum and maximum potential compatibility of two random phenotypes for a given trait-adjacency matrix; this is done by comparing 10^6^ pairs of phenotypes generated at random according to the same criteria used in the virtual species-generation process).

The concept behind this approach is well-grounded in theoretical ecology because it corresponds to generating an ideal global square matrix of species-by-species compatibility [e.g., similar to stability-complexity matrices (*43*)]. Adding explicit traits from another matrix of trait-by-trait compatibility adds realism by ensuring that phenotypic compatibility is spatially and temporally consistent. It also reduces the computational burden by providing an efficient way to assess compatibility of any two species in the world without needing to retain a large compatibility matrix; the approach also provides different degrees of specialization (as observed in real-world networks). Although this approach precludes comparing our functional traits to real-world traits, the mechanism produces realistic networks (fig. S4). The final step in the generation of a virtual species requires attributing a random real value between 0 and 1 to it to indicate its adaptive capacity to changing conditions (see the “Adaptation” section below and fig. S23).

### Populating a virtual Earth

We obtained land-use projections at annual temporal resolution from 2006 to 2100 from Chini *et al.* (*44*). We used projections calibrated using Integrated Assessment Model scenarios with radiative forcing by 2100, consistent with the SSP scenarios used for climate projections—4.5 W m^−2^, 6 W m^−2^, and 8.5 W m^−2^. The original data were at a resolution of 0.5° × 0.5°, which we rescaled to our 1° × 1° grid. We did not consider all land-use categories; instead, we computed for each cell in our grid the fraction of primary and secondary land in a given year. We report the loss of primary and secondary land by 2050 and 2100 under the different climatic scenarios (fig. S24). Here, “primary” land is defined as natural vegetation that has never been affected by humans (e.g., agriculture or wood harvesting), while secondary land is natural vegetation recovering from previous anthropogenic disturbance (*44*).

We assigned 4500 × *u* species to each locality at random, where *u* is the proportion of primary + secondary land in the locality according to the target SSP scenario in 2019. We then reduced the local community to the set of species capable of persisting in the local climate as all those species for which the combined probability of extinction under local temperature and precipitation conditions is < 0.05 for the entire period 2015–2019 (with probability computed monthly). Last, we arranged these species into food webs (see the next section), keeping only the species participating in the network.

### Building networks

For a given pool of species, all potential pairwise interactions are considered, and a directional link is drawn from a candidate resource to a candidate consumer if (*i*) the trophic level of the consumer is greater than the trophic level of the resource, and the trophic level of the resource is within the trophic breadth of the consumer (i.e., not less than the minimum trophic level of a species possibly consumed by the consumer); (*ii*) the ratio between the body size of the resource and that of the consumer is greater than *l_l_* < *l*_u_, with those limits assigned specifically to each combination of resource-consumer taxa (i.e., birds consumed by mammals are assigned different values than birds consumed by reptiles); and (*iii*) functional compatibility (*c*_f_; see above) is > 0.55. To obtain *l_l_* and *l*_u_, we computed the ratio between prey and predator body mass for each prey-predator interaction recorded in *GloBI* (*42*); this provided a distribution of prey-predator body-mass ratios. We then derived *l_l_* and *l*_u_ as the lower and upper 90% confidence limits of the distribution, respectively. We applied this procedure separately for each combination of vertebrate classes, such that we obtained a distribution of prey-predator body mass ratios for mammals eating birds, another one for birds eating mammals, another one for mammals eating reptiles, etc.

Functional compatibility is then readjusted to $c_{f}=1-\frac{1-c_{f}}{1-0.55}$ and assigned as a weight to the target link. We chose the functional compatibility threshold (*c*_f_ = 0.55) and the number of random species assigned to each locality (4500) after calibration experiments as the best values permitting realism in terms of local diversity and network structure (figs. S1 to S4). See also low sensitivity of model outputs to variation in *c*_f_ (Supplementary Materials).

### Colonization

At each step of the simulation, a species attempts dispersal from each locality to neighboring localities. A distance (in degrees) of dispersal is first randomly extracted from a log-Normal distribution with mean = 0 and SD = 1 (fig. S25). We added 1 to that distance to ensure that in the shortest-distance case, a species can still move to an adjacent cell in the grid. One cell in the grid at the randomly selected dispersal distance is chosen at random. Then, the potential colonizer species moves to the target locality with a probability computed by testing a species’ niche against the climatic condition of the target locality (in the same way as extinction probability is evaluated).

Such a basic treatment of movement of species from one locality to another could conceivably increase diversity indefinitely. However, in the coextinction scenario where we modeled food webs explicitly, the process is realistically constrained because species need to find their place in the food web that is conducive to survival. This is modeled so that when a new species enters a community based solely on climate compatibility, the local food web is rebuilt (according to the same rules described above), taking the new species into account. This might lead to different outcomes in each case.

For example, if the candidate colonizer is not a primary consumer and cannot find suitable resources, it cannot enter the food web, and colonization fails (i.e., it goes locally extinct). If a candidate colonizer does find a position in the food web, it can become associated with some resources and possibly become a resource for local consumers itself without driving those resources to extinction. Alternatively, if a candidate colonizer finds a position in the food web but ends up overexploiting one or more resources, it can lead to extinction of the resources and potentially itself and other species via extinction cascades.

However, if a candidate colonizer is a “primary” consumer (i.e., a vertebrate capable of consuming plants and/or invertebrates), it will always be able to find a position in the food web and become a resource for local consumers, producing an ecologically unrealistic accumulation of diversity and resources. This is because of the specific architecture of the model, where we assumed herbivores and insectivores are the “basal” components of the networks (i.e., we assumed continued and unlimited availability of their resources). For this reason, we treated the incidences of insectivores and herbivores moving to a new locality as a particular case. Here, candidate herbivore and insectivore colonizers are added to the local community up to a maximum number of species, defined as the initial herbivore/insectivore diversity for that locality. If the diversity of insectivores and herbivores exceeds that number, the (herbivore and/or insectivore) species with least compatibility to local conditions are removed (i.e., considered as outcompeted by the other species).

In the scenario not accounting for coextinctions (i.e., not modeling food webs explicitly), we applied the same criterion but extended to all species in the locality, regardless of their trophic ecology (with the only other criterion for colonization success being climatic compatibility) (see the Supplementary Materials for caveats). In this way, we treat the null expectation of primary extinctions only as a conservative case.

### Adaptation

We assumed that species have a certain capacity to adapt to local (and changing) climates unrelated to the traits defining phenotype. For this, each species has the opportunity with probability *p*_adp_ = 0.001 of shifting the center of its niche closer to the average local conditions at yearly intervals (i.e., a species during year *y* can move its niche to fit conditions recorded in year *y*^−1^). Here, both the average and upper/lower tolerance limits for both temperature and precipitation are shifted along the segment connecting the center of the species’ niche and the mean local temperature and precipitation of the preceding year at a length given by the formula *C*_adp_ × *A* × *d*_N_, where *C*_adp_ is an overall simulation adaptation factor that we set to 0.01 (fixed for all simulations), *A* is species-specific adaptability (a random value between 0 and 1 assigned when creating the virtual species), and *d*_N_ is the total distance between the center of the species’ niche and mean local conditions (see fig. S26). After that, the niche is recomputed on the basis of the updated parameters as described in the “Species climatic niche” section.

We also explored the sensitivity of results to the choice of the adaptation parameters *p*_adp_ and *C*_adp_ (see the “Sensitivity analyses” section and figs. S6 and S7). Our goal in implementing adaptation was not to model this process explicitly, which would be unfeasible due to the countless unknowns associated with the process. Rather, we wanted to increase the conservativeness of our model by providing species and communities with an additional mechanism to cope with climate change. Such a mechanism mimics local acclimation (with species shifting their niches to match local climate, as described in fig. S26) and does not necessarily entail any evolutionary process.

### Coextinction

We developed an original, intuitive model for simulating coextinctions that accounts for both bottom-up and top-down effects of diversity loss. The model is applied to weighted, directed networks (with weights corresponding to species’ functional compatibility; see the previous section). Such networks can be represented in the form of a square adjacency matrix **A** of size *n* × *n* (where *n* is the total number of species in the network), with each entry **A**_*i*,*j*_ (with *i*,*j* in [1, …, *n*]) representing the interaction strength between the *i*^th^ resource and the *j*^th^ consumer. Thus, Σ(**A**_*i*,1:*n*_) represents the total consumer pressure on resource *i*. Each value in row *i* is divided by the total consumer pressure and then multiplied by its original value to obtain a standardized weight accounting for competition for that resource. For example, if one resource is used by three consumers with respective weights 0.5, 0.2, and 0.1, the rescaled values are as follows: 0.5 × (0.5/0.8) = 0.3125, 0.2 × (0.2/0.8) = 0.050, and 0.1 × (0.1/0.8) = 0.0125.

The overall idea behind this step is to account for both the overall competition on a certain resource and resource-consumer compatibility. Let us call the rescaled matrix **A**′. The column sum of the rescaled values, Σ(**A**′_*1*:*n*,*j*_), gives the amount of resource available to consumer *j*. If that amount falls below a certain threshold, the consumer goes extinct. Similarly, the row sum of the rescaled values, Σ(**A**′_*i*,*1*:*n*_), indicates the pressure of consumers on resource *i*. Should this pressure go beyond a given threshold (see below), then resource *i* goes extinct due to overconsumption.

Species can also go extinct whenever they become disconnected from basal resources (in our case, corresponding to being detached from herbivores or insectivores). For each network, thresholds for consumer extinctions correspond to the minimum observed Σ(**A**′_*1*:*n*,*j*_) for any species *i*,*j* in the network at the time the network is generated. Similarly, thresholds for extinctions of resources following overconsumption are set to the maximum observed Σ(**A**′_*i*,*1*:*n*_) for any species *i*,*j* in the network at the time the network is generated. The thresholds are then updated after the burn-in phase (see the “Simulations” section) to ensure that networks are at equilibrium once the global-change simulation starts, and coextinction events can only be generated by removing some species from the network. Our initial, conservative assumption here is that no species goes extinct if conditions do not change (i.e., if environmental conditions remain stable and no species immigrate from different locations). However, this does not prevent new species from entering the system and increasing local diversity. This assumption also necessarily gives a conservative estimate of total extinction rates given that our model does not explicitly incorporate existing extinction lags before each simulation beginning (*45*).

The threshold for consumer coextinctions is assigned specifically to each network by referring to the most exploited resource in the system. This conservative assumption provides substantial opportunity increase in consumer diversity (following species immigration from other localities) before top-down coextinctions are triggered. The top-down regulation mechanism serves two main purposes: (*i*) It accounts for the potential detrimental effects of alien colonizers on both native resources and potential competitors (with outcompetition processes treated with specific model rules; see the “Colonization” section),and (*ii*) it accounts for decreasing resource diversity in a food web eliciting two different, concomitant effects: less-efficient consumers might be outcompeted by more efficient ones, and resources targeted by many different consumers might be overexploited. There are clearly ecological settings where these processes can be buffered or even reversed, but the rapid environmental and ecological change we modeled here is more likely to destabilize than to stabilize systems, consistent with increasing empirical evidence of ongoing global ecosystem collapse (*2*–*5*, *46*).

Our dynamic implementation of network weights also compensates for the choice (dictated by the insurmountable computational challenge of modeling multispecies population dynamics for almost 20,000 networks at a temporal span of nearly a century) of not tracking explicitly species abundances. Our implementation of weights is conceptually linked to species abundances in that the total interaction weight of a consumer (which eventually determines its risk of going extinct following ecological change in the community/food web) increases with the availability of suitable resources and declines with diversity of competitors. At the same time, the total pressure on a resource determining its extinction risk increases with diversity of (compatible) consumers. It is reasonable to assume that when the consumer’s population has access to a large variety of suitable resources and it has few competitors, it will be larger than a population able to access fewer resources shared among many consumers. Similarly, the population of a resource (and hence, its availability to consumers) will decrease with increasing consumer pressure. It is therefore also reasonable to consider weights in our networks as a proxy for abundances, accounting for multispecies population dynamics in the global model without explicitly implementing them.

### Simulations

We ran 100 simulations per climate-projection scenario. In each simulation, we generated a pool of species, and we populated a virtual Earth as described in previous sections. We did a burn-in phase where we permitted species to disperse to new areas over 100 time steps. At each step of the burn-in and for each locality, we sampled one species and moved it to a randomly selected locality following a dispersal kernel of the form 1 + log𝒩 [0,1]. We then evaluated a species’ survival probability under the climate conditions from 2015 to 2020, keeping it in the simulation only if the probability of extinction *p*_ext_ < 0.05. We rebuilt food webs every 10 steps. If the number of basal species increased because of colonizers exceeding the initial number of basal species (i.e., at the beginning of the simulation), we evaluated climatic suitability of all basal species, removing species in increasing order of local adaptation until we restored the initial number of basal species. The burn-in phase provided our simulated systems with biogeographical realism, giving species a chance to colonize contiguous areas if climatically suitable (and within a food web permitting the colonization). In addition, it increased the robustness of networks to local, current (2015–2020) climatic conditions through the potential replacement of basal species with better-adapted colonizers. Last, it ensures that the initial changes recorded in the model run are not biased by the short-range movements of species from the initial random pools assigned to each locality.

We then simulated climate and land-use changes from 2020 to 2100. At monthly steps and for each locality, we evaluated which species went extinct according to their suitability to withstand local temperature and precipitation conditions. We considered that species with *p*_ext_ ≤ 0.05 never went extinct (we used this probability to assign species to localities). In this way, we avoided “random” extinctions, such that extinctions only occurred because of climate and/or land-use change. We also assumed that in each year and in a given locality, a fraction of the species identical to the fraction of primary and secondary land lost from the previous year went extinct (see the next paragraph for additional details). Last, we simulated coextinctions in food webs due to primary extinctions and then updated the networks. We replicated the same simulations in a control scenario not accounting for networks and secondary extinctions. The two scenarios (coextinction and control) started from the same initial virtual Earth (after the burn-in, predispersal/colonization phase) but then ran each independently.

The choice of using a monthly time step for climate change and yearly time steps for the land-use change was driven by the different temporal resolutions of the selected dataset. However, the different temporal resolutions are also well-suited to model the two different processes, considering the potential high variability of temperature and precipitation patterns on a relatively short time scale compared to the slower and usually nonreversible (at least in the short term) losses of primary land.

### Effect of land-use change on species diversity

Land use change might have complex and case-specific effects on vertebrate communities. The response of local vertebrate diversity to the intensity of land-use change—measured as the fraction of primary and secondary land lost at each yearly step in the simulation compared to the previous year—might assume different shapes. Those ideally range from cases where small land-use changes result in disproportionately high diversity loss to the opposite where diversity persists in the face of severe losses of primary and/or secondary habitat. We opted for an intermediate scenario where the loss of species diversity is linearly proportional to land conversion. However, we also explored the potential effects on model outcomes of simulating different forms and magnitudes of response of local diversity to land use change.

Specifically, we explored the potential impact on results of using different response curves of biodiversity loss versus relative land-use change of the following form

$$loss=1-{\left(1-P_{LUC}\right)}^{R_{1}}\;\mathrm{if}\;R_{2}=-1,\;\mathrm{or}\;loss=P_{LUC}^{R_{1}}\;\mathrm{if}\;R_{2}=1$$

where *loss* is the fraction of biodiversity lost following habitat loss via land-use change; *P*_LUC_ is the fraction of primary and secondary land lost at a given time step compared to the previous one; *R*_1_ is a random number in the range {0,1}; and *R*_2_ is sampled from {−1,1} with equal probability.

The two parameters *R*_1_ and *R*_2_ can be combined into a single parameter *S*_LUC_ = *R*_2_(1 − *R*_1_), which describes the response curves of diversity loss in a continuous way from 1 (full diversity loss occurs even with no habitat loss) to −1 (diversity loss is invariant to full habitat loss), with *S*_LUC_ = 0 representing the linear response we used in the main simulations (fig. S27).

The effect of land-use change could also affect species differently depending on body size and trophic level (themselves partially dependent properties). However, the mechanisms by which the impact of land-use change might reach different species in a community could be complex. Our model already accounts for such complexity. The expectation that large-bodied animals are more susceptible to the effects of climate and land use change results from observation of patterns, but the underlying mechanisms are unclear. This is where our model offers another unique advantage compared to existing studies—climate and land-use change are predicted to cause a global reduction in vertebrate body size (fig. S11) even without any initial assumption of an intrinsic relationship between body size and species vulnerability. That is, our model offers a mechanistic explanation of the process—body size reduction is mainly a consequence of how diversity loss propagates through food webs. That this well-known pattern arises naturally as an emergent property of “simple” rules provides strong additional support of our model’s ecological realism. Nevertheless, we also explored the potential impact on our results of assuming an a priori relationship between vertebrate body sizes and vulnerability to land-use change in the sensitivity analyses (see below), devising a procedure controlled by a single parameter (*V*_LUC_) as follows: Consider a local community with *S* species—at a given step, *loss* × *S* species will go extinct due to land-use change (with *loss* being the fraction of biodiversity lost following habitat loss via land-use change, computed as described above). To identify such species within the local species pool, we first sorted species by decreasing size. We then iterated the procedure of randomly switching the position of two species in the ordered list *V*_LUC_ × *S* times. Last, we identified the first *loss* × *S* species in the sorted list as extinct. When *V*_LUC_ = 0, the largest species always go extinct due to land-use change at each step, while increasing *V*_LUC_ leads progressively toward random extinctions (fig. S28).

### Sensitivity analyses

We explored the potential sensitivity of our model outputs to different combinations of parameter values by devising a global sensitivity analysis where we modified (*i*) the number of steps in the burn-in phase (*s*_b-i_), (*ii*) the threshold for functional compatibility (*c*_f_), (*iii*) species adaptation probability (*p*_adp_), (*iv*) species adaptation factor (*C*_adp_), (*v*) the shape of the response curves of local diversity versus land-use change (*S*_LUC_), and (*vi*) the potential relationship between vertebrate body mass and vulnerability to land-use change (*V*_LUC_). Here, we ran an additional set of 100 simulations per future climatic scenario where we randomly sampled (uniformly) *s*_b-i_ from 0 to 1000, *c*_f_ from 0.45 to 0.65, *p*_adp_ from 0 to 0.005, *C*_adp_ from 0 to 0.05, *S*_LUC_ from −1 to 1, and *V*_LUC_ from 0 to 1. For each simulation, we recorded the percentage loss of diversity as the response for each climate change scenario. We then applied a machine-learning emulator—boosted-regression trees (*47*)—implemented using the dismo R library (*48*) and its function *gbm.step* to examine the relative importance of each of the four variables considered on the response. We set the error distribution family as Gaussian, the bag fraction to 0.75, the learning rate to 0.001, the tolerance to 0.0001, and the tree complexity to 2 (i.e., first-order interactions only). To assess the relative effect of each variable on initial diversity, we calculated the boosted regression tree metrics of relative influence (relative influence *I* is defined as the relative influences of the individual inputs *x_j_* on the variation of the function that maps the explanatory variables *x* to the response variable *y*) (*49*).

## Supplementary Materials

### This PDF file includes:

Supplementary Text Figs. S1 to S29 Table S1 References
