# Supplementary material for: Coextinctions dominate future vertebrate losses from climate and land use change
Source: Sci Adv. 2022 Dec 16;8(50):eabn4345. doi: 10.1126/sciadv.abn4345 (PMC9757742; doi:10.1126/sciadv.abn4345)
Supplement: Supplementary file 1 — Supplementary Text Figs. S1 to S29 Table S1 References [file sciadv.abn4345_sm.pdf]

Science Advances
AAAS

Supplementary Materials for

Coextinctions dominate future vertebrate losses from climate and land use change

Giovanni Strona and Corey J. A. Bradshaw

Corresponding author: Giovanni Strona, giovanni.strona@ec.europa.eu

Sci. Adv. **8**, eabn4345 (2022)
DOI: 10.1126/sciadv.abn4345

This PDF file includes:

Supplementary Text
Figs. S1 to S29
Table S1
References

Supplementary Text

Network realism

We compared our networks with a large dataset of food webs to check their consistency in terms of node density and diameter. When generating food webs by using ecological proxies, one risk is creating networks that have an unrealistically high density of links (and hence, a short diameter due to the abundance of paths connecting each node to any other node in the network) (*50*). In our method to generate food webs, we established several steps to prevent such issues. We tested a random sample of 1000 networks per climatic scenario with a large dataset including 358 real-world food webs (globalwebdb.com). Our food webs have a realistic structure in terms of connectance (number of edges ÷ squared number of nodes; Fig. S4a) and diameter (Fig. S4b).

Diversity losses versus *gains*

Climate change elicits both 'winning' and 'losing' species; that is, while we expect many (likely most) species to experience range contractions, or local and/or even global extinctions, we can also expect many species to experience new opportunities for colonization and range expansions (*51, 52*). Our model allows for such phenomena due to the implemented mechanisms of dispersal, colonization, and adaptation. A colonizer disperses according to the dispersal kernel, and once in the target locality, it is subjected to climate constraints in both scenarios; that is, it will go extinct in the next step with a probability given by the compatibility between its niche and local climate (it might also go extinct due to land-use change as for any other species). Colonizers not immediately committed to extinction by climate or land-use change might drive other species to extinction (see section regarding overexploitation in the co-extinction model), and/or serve as an additional resource for other species, or drop out of the network altogether if not able to access resources (regardless of climate).

We assumed that basal species (herbivores and insectivores, i.e., consumers and not primary producers) are exploiting all available resources at equilibrium; thus, basal species can enter the network at any time. But when the number of basal species in the system exceeds the starting values (i.e., number of basal species present in the target locality at the beginning of the simulation: 2015), the adaptation to local climate conditions of all the species in the target network/community is re-evaluated, and then basal species are removed from the least- to the most-adapted until their number returns to the initial value. This rule prevents basal species from being added indefinitely to the network depending on simple climate compatibility, which would unrealistically increase diversity and network robustness indefinitely. Conversely, non-basal species able to find a niche in the network and that are compatible with local climate conditions can be added indefinitely to the network. This

is because potential overexploitation of resources is implemented in the co-extinction model and hence, it prevents unrealistic scenarios where the increased diversity is not supported by available resources (i.e., there is too much competition for a given resource).

In the control scenario, we did not code for the network rules determining the success of colonizers, so we had to make an explicit rule to avoid unrealistic increases in diversity arising from too many species colonizing. As in the co-extinction scenario, we assumed starting (2015) communities to be at energetic equilibrium. Thus, we assumed that diversity could not increase and that in case of colonizers entering a system at full capacity (i.e., with same number of species as in 2015), we needed to implement the same procedure as described above (ranking species according to local climatic adaptation and then removing the less-adapted species) to restore the initial diversity.

This means that there are possible situations where at a given time step, diversity in the co-extinction scenario can be paradoxically higher than the diversity in the control. This is an obvious consequence of the different model designs (which, in turn, represent the best compromise we found to overcome the challenge of making virtual worlds with and without ecological interactions comparable) when evaluating/comparing relative diversity loss between the control and the co-extinction scenarios; we scaled the control diversity in those cases (a conservative choice in our evaluation of global patterns of diversity loss). In addition, gains in diversity are an interesting aspect *per se*, but the focus of our paper is diversity loss and the amplifying effect of co-extinctions. We therefore calculated percentage losses assuming an increase in diversity as a zero loss. Most gains in diversity occurred in areas of initial low diversity and consist usually of a net increase in diversity of one or a few species only (Fig. S29).

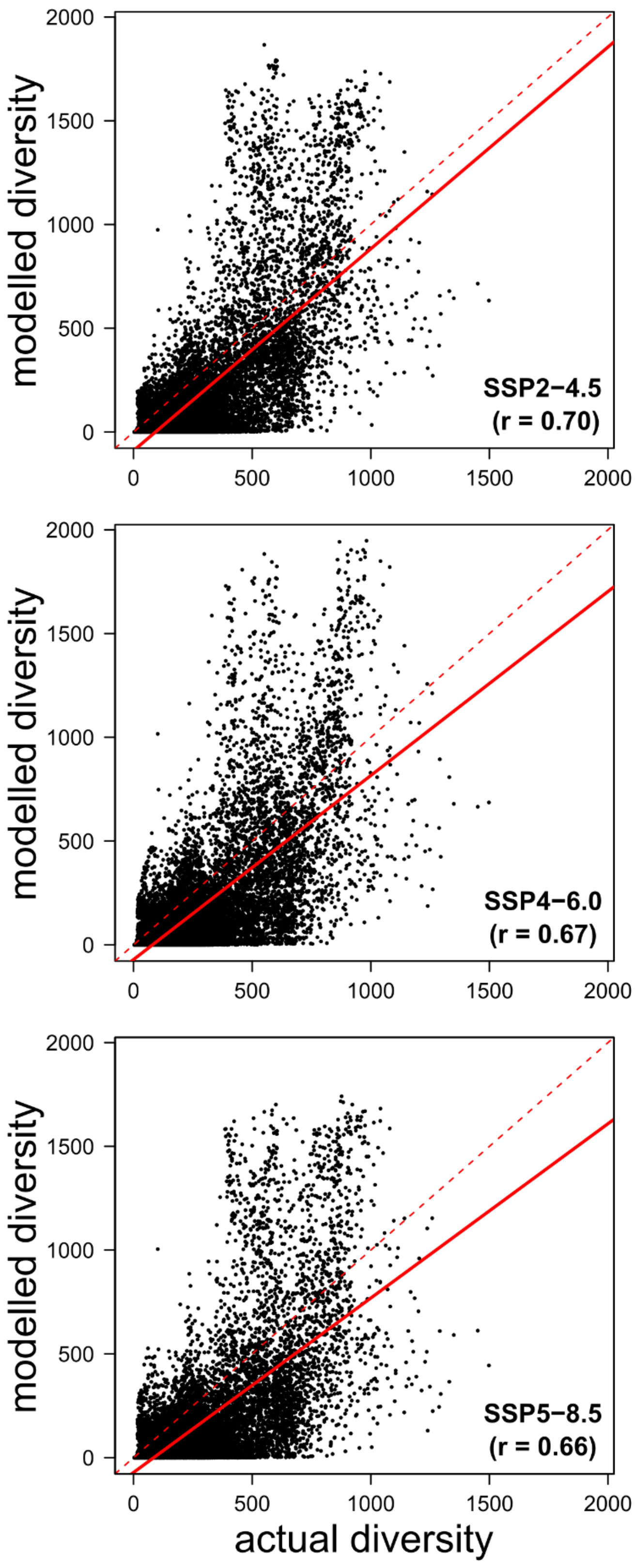

Fig. S1. Comparing measured diversity (computed as cumulative number of species having range intersecting in a given 1×1° latitude cell, using all IUCN and BirdLife species ranges) and simulated diversity in the different climate scenarios for 2015. Solid line is the least-squares regression and dashed line indicates 1:1 relationship. Equations for least-squares regression are: SSP2-4.5: $y = -89.46 + 0.97x$; SSP4-6.0: $y = -70.65 + 0.89x$; SSP5-8.5: $y = -71.20 + 0.84x$. Sample size is 17334 in all plots.

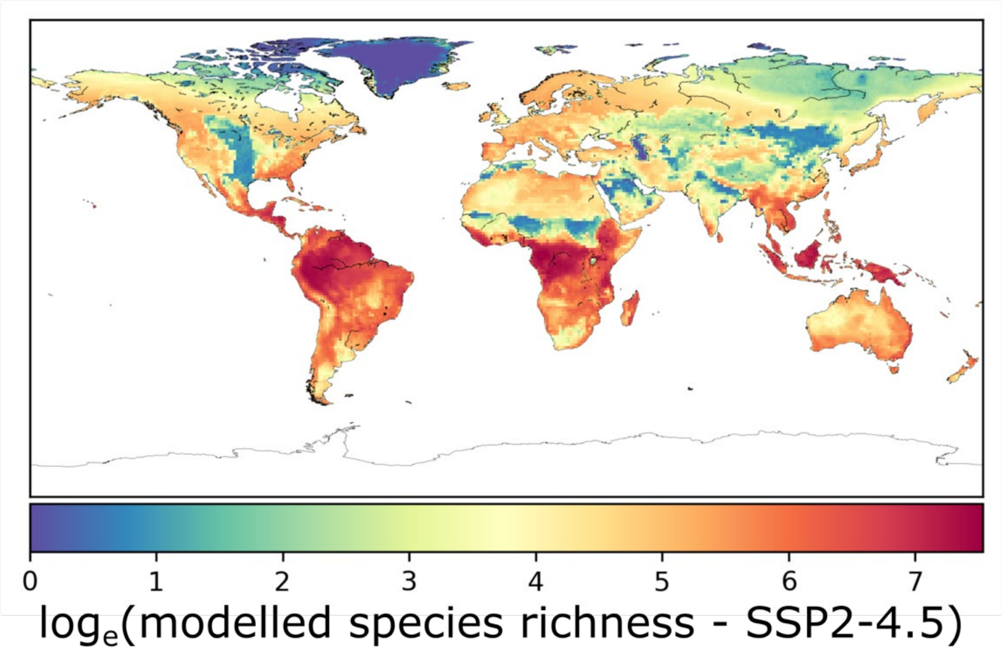

Fig. S2. Modelled vertebrate diversity in 2020 in the CMIP6 SSP2-4.5 scenario. Local diversity (quantified as total number of species per locality) averaged across 100 model replicates (resolution is 1×1°).

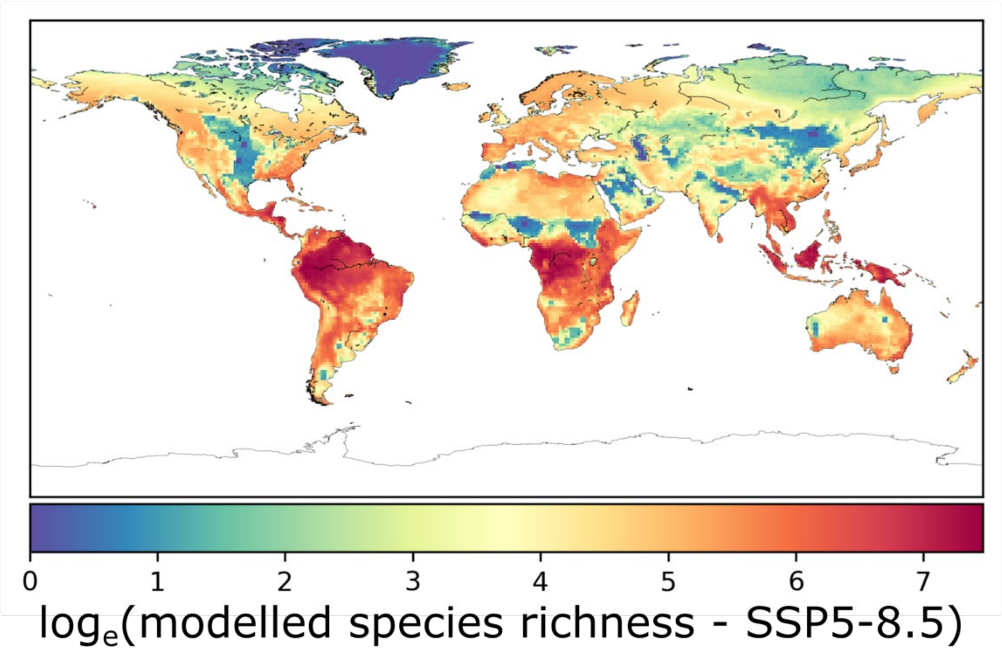

Fig. S3. Modelled vertebrate diversity in 2020 in the CMIP6 SSP5-8.5 scenario. Local diversity (quantified as total number of species per locality) averaged across 100 model replicates (resolution is 1×1°).

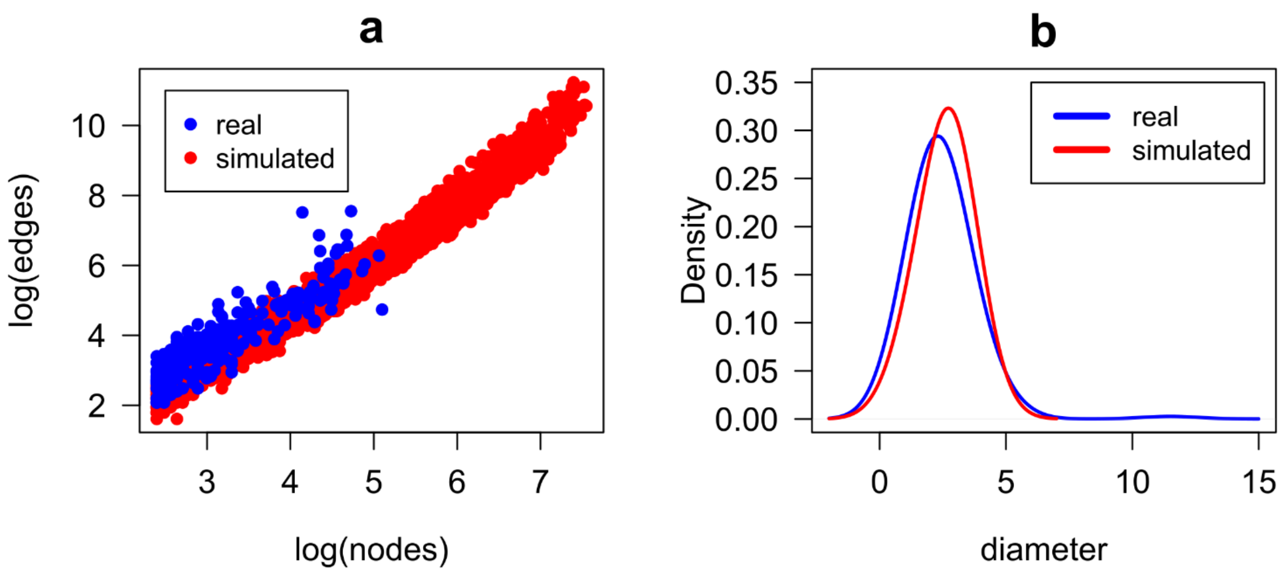

Fig. S4. Comparing basic structural properties of the simulated networks and a set of real-world food webs. We compared a random sample of 1000 simulated networks per climate-projection scenario, with a large dataset including 45 real-world terrestrial food webs (from globalwebdb.com). (a) Relationship between node and edge number shows that connectance in the two sets of networks scales consistently with network size. (b) Distribution density of network (directed) diameter in real *versus* simulated networks. Network diameter is the longest among all shortest paths between any pair of nodes in the network, hence representing a measure of trophic complexity in a food web.

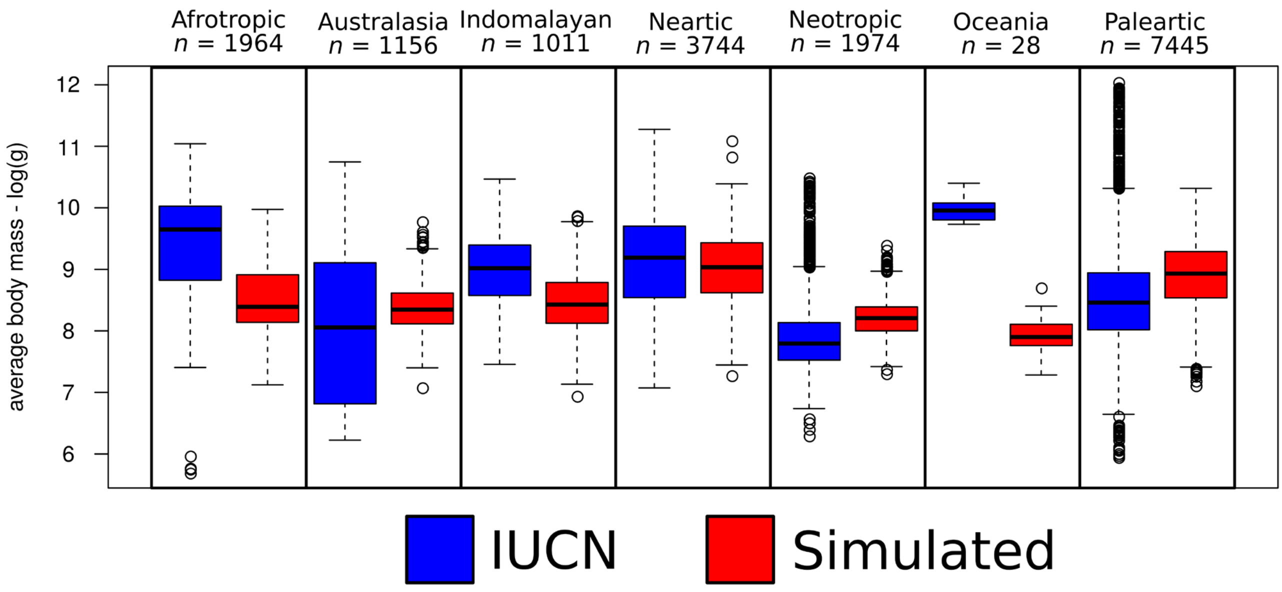

Fig. S5. Modeled vs. observed regional distribution of vertebrate body masses. Distribution of average vertebrate body mass across all localities within different biogeographical regions obtained from combining IUCN vertebrate ranges with our dataset of vertebrate body masses (blue; see Methods) and from one randomly selected replicated world generated by our model (red). Numbers indicate the number of localities (1×1° cells) for each region. Boxes indicate first and third quartiles, whiskers indicate range, horizontal lines indicate medians, and dots indicate outliers.

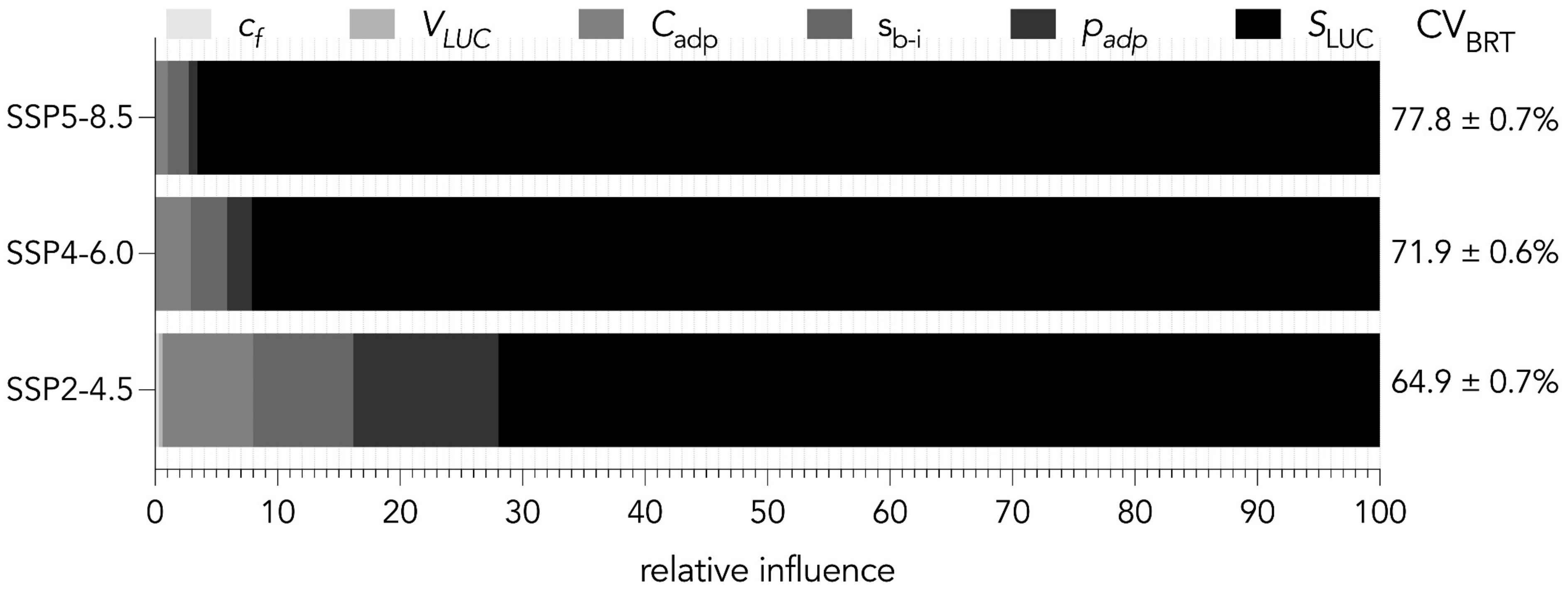

Fig. S6. Relative influence of variation in six model parameters on diversity loss. Based on a global sensitivity analyses using a boosted regression-tree emulator (see main text Methods), we tested the influence of variation in (*i*) threshold for functional compatibility c_f, (*ii*) relationship between vertebrate body mass and vulnerability to land-use change V_{LUC}, (*iii*) species adaptation factor C_{adp}, (*iv*) number of steps in the burn-in phase $s_{b\text{-}i}$, (*v*) species adaptation probability p_{adp}, and (*vi*) shape of the response curves of local diversity vs. land-use change S_{LUC}. The most influential parameter was S_{LUC} among all climate-change scenarios (SSP2-4.5, SSP4-6.0, SSP5-8.5 — see main text for scenario descriptions), with relative minor contributions of C_{adp}, p_{adp}, and $s_{b\text{-}i}$; variation in c_f and V_{LUC} had almost no effect on predictions of diversity loss. Also shown are the boosted regression trees goodness-of-fit metrics (coefficient of variation ± standard error).

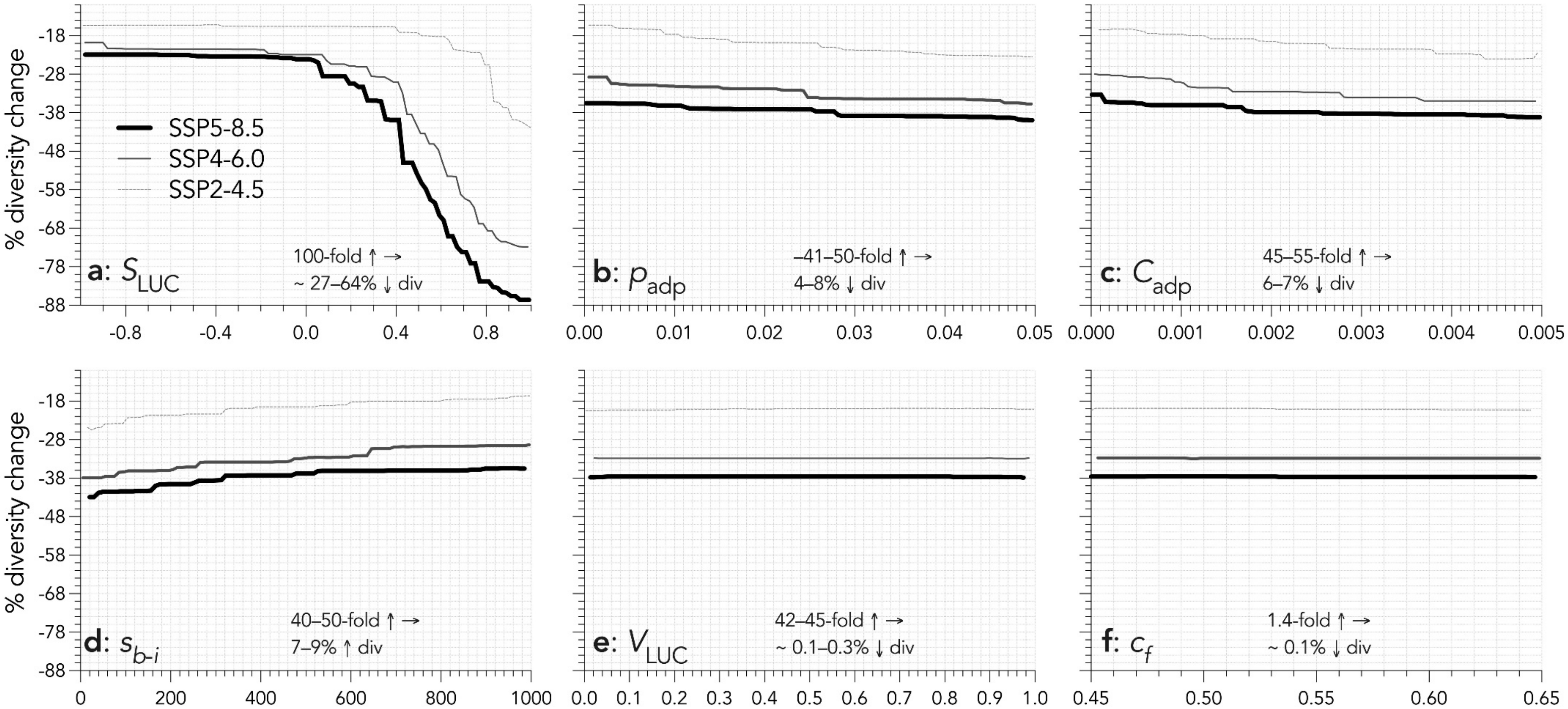

Fig. S7. Boosted-regression tree results from the global sensitivity analyses showing the direction and magnitude of the relationships between the modelled response of % diversity change and variation in six modelled parameters. Relationships between % loss of diversity and variation in six model parameters: **a** shape of the response curves of local diversity vs. land-use change S_{LUC}, **b** species adaptation probability p_{adp}, **c** species adaptation frequency C_{adp}, **d** number of steps in the burn-in phase s_{b-i}, **e** relationship between vertebrate body mass and vulnerability to land-use change V_{LUC}, and **f** threshold for functional compatibility c_f. The relationships for the three climate-change scenarios considered (SSP2-4.5, SSP4-6.0, SSP5-8.5 — see main text for scenario descriptions) are shown for each parameter. Also shown in each panel is the approximate range of percentage change in the response variable (% change in diversity loss) relative to a *x*-fold range change in the model parameter.

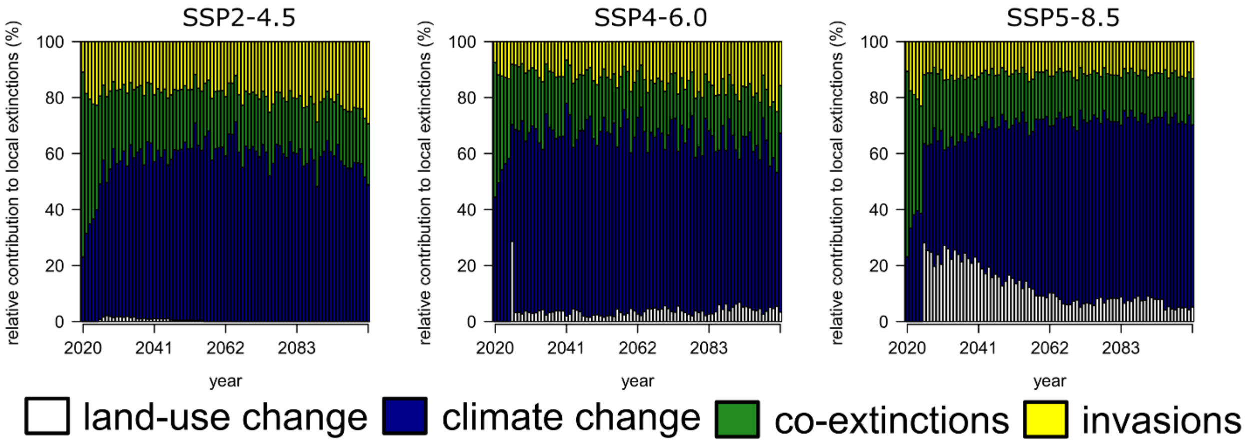

Fig. S8. Relative contribution of different extinction drivers to species loss. Relative yearly percentage of total recorded local extinction events (averaged across 100 simulations) for each extinction driver. Invasions refer to the replacement of a local species by a colonizer from a different locality.

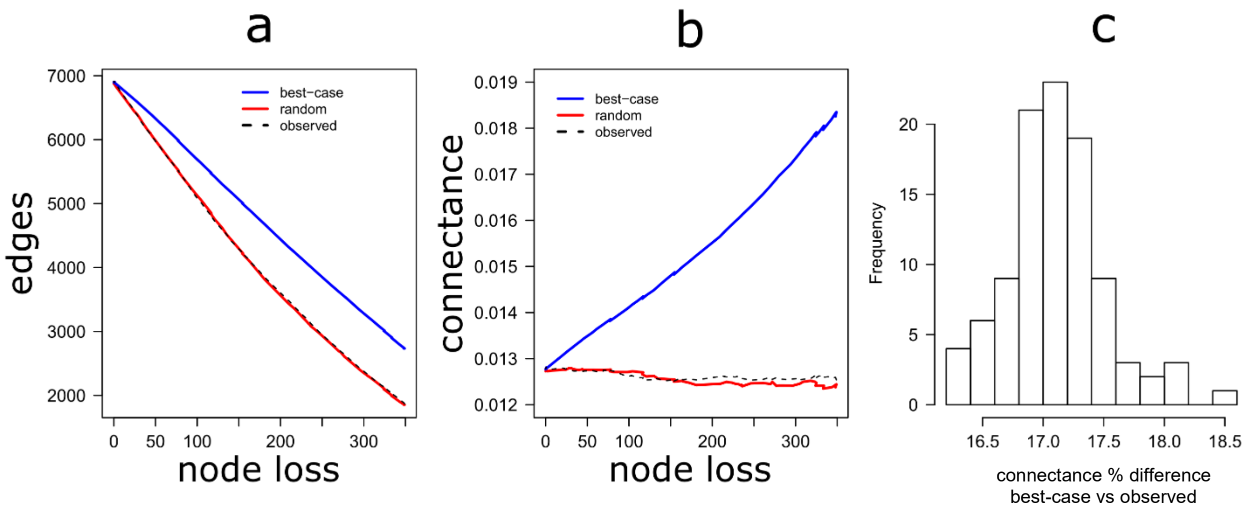

Fig. S9. Example of different trajectories of edge loss and the resulting changes in network connectance following primary and secondary extinctions. We randomly selected one simulation (for the intermediate emission scenario SSP4-6.0) and, from that, we selected 100 networks from randomly chosen localities (in 2020). We then disassembled those networks by progressively removing a total number of nodes identical to the number of species lost in the target network during the simulation according to two different criteria, keeping track of the resulting changes in the number of edges (**a**) and network connectance (**b**). Specifically, we either removed nodes randomly (red lines), or in decreasing order of their degree (best-case; blue lines). We compared the trajectories of change in number of edges (**a**) and connectance (**b**) with the actual corresponding values recorded during the global extinction simulations (dashed lines). Panel **c** reports the frequency of the percentage difference in the final connectance obtained from the best-case scenarios and the final connectance recorded in the actual global extinction simulations. This shows how the observed decline of connectance is substantially higher than that would be expected under a best-case scenario, which corresponds to the background expectation in lack of global environmental change (*30*).

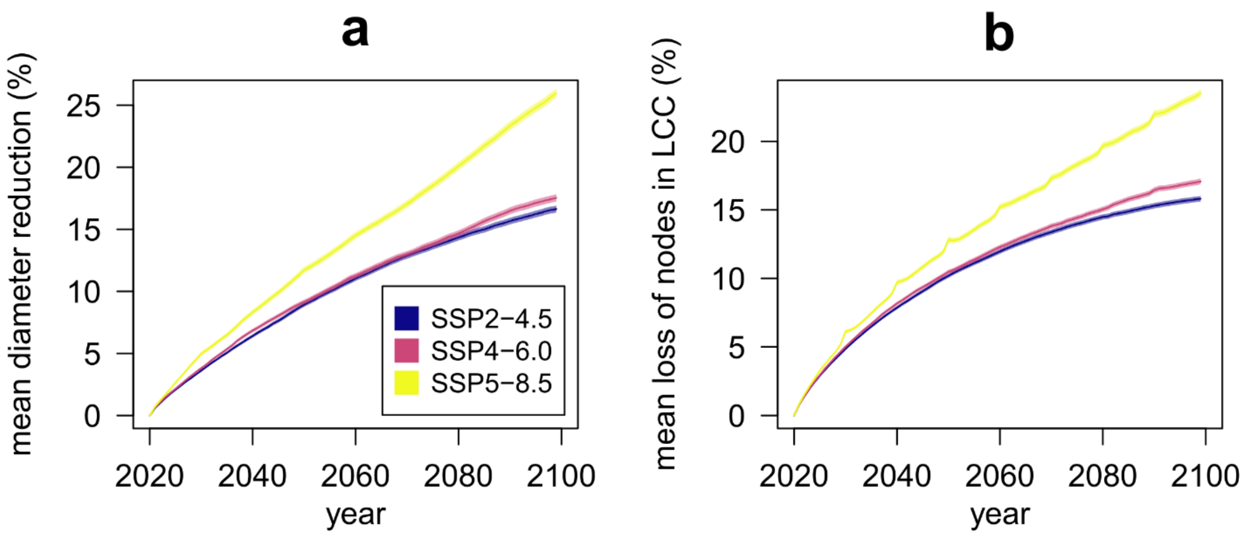

Fig. S10. Future projections of structural change of networks under the different climate-change projections. In the worst-case climate-change scenario (SSP5-8.5), we expect an average reduction of (**a**) network diameter and (**b**) of the fraction of nodes in the largest, most weakly connected network component (LCC, which is the largest cluster of nodes for which a path to at least another node in the network exists) of almost 25% compared to the reference local conditions in 2020. Solid lines represent the average of 100 simulations (per climate-projection scenario). We first averaged local loss in the 100 replicates, and then averaged local loss values across all localities. Shaded areas are 95% confidence intervals, showing the variation across localities in the average map obtained with 100 simulations.

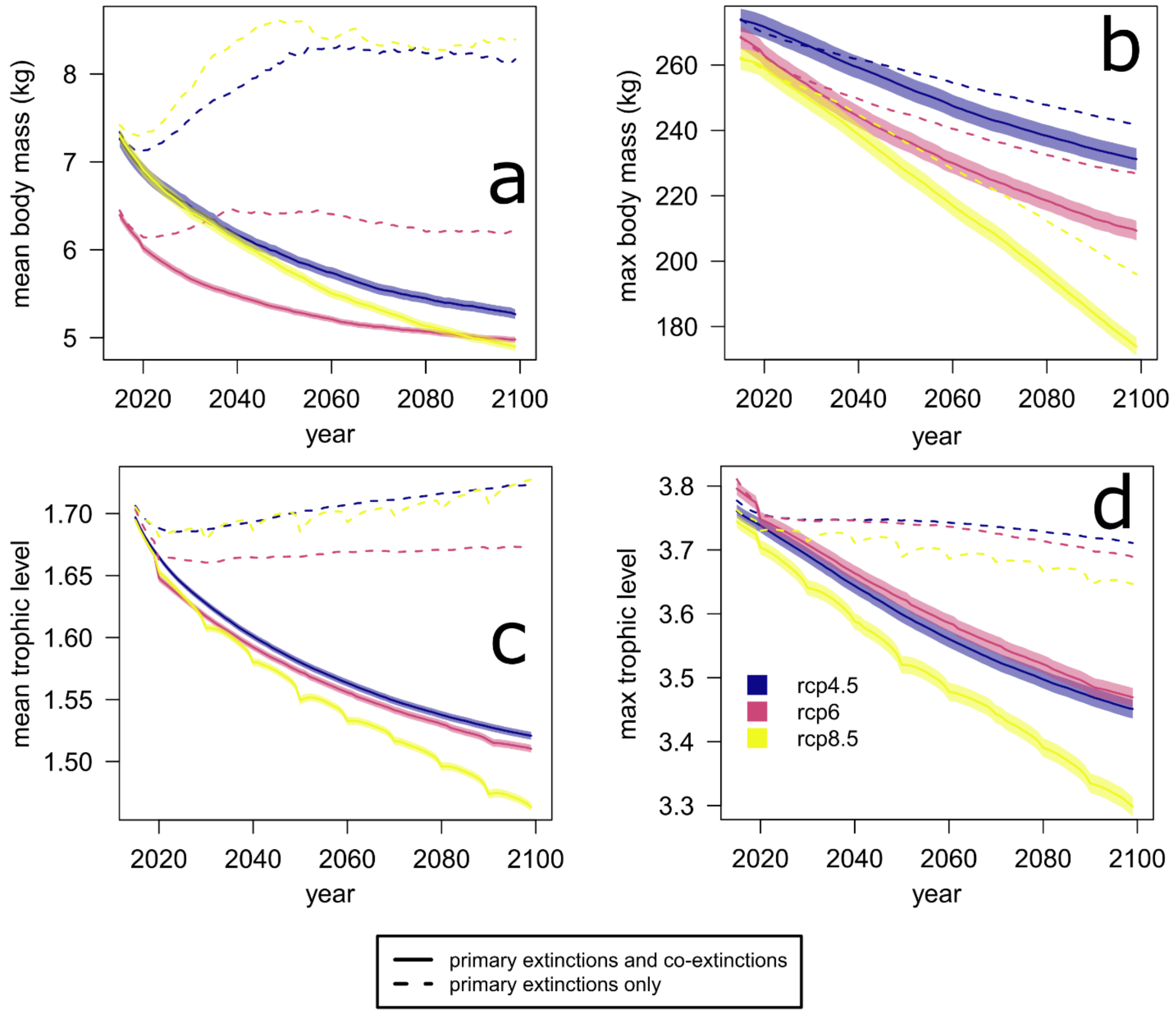

Fig. S11. Variation in average and maximum local trophic level and vertebrate body mass under different climatic projections from 2015 to 2100. Average of 100 replicates (we averaged values for the target variable in each locality across 100 replicates, and then averaged local values across all localities for a given year). Shaded areas are 95% confidence intervals, showing the variation across localities. Dashed lines represent the averaged local values of the target variables obtained from the control simulations (i.e., those where we did not simulate co-extinctions).

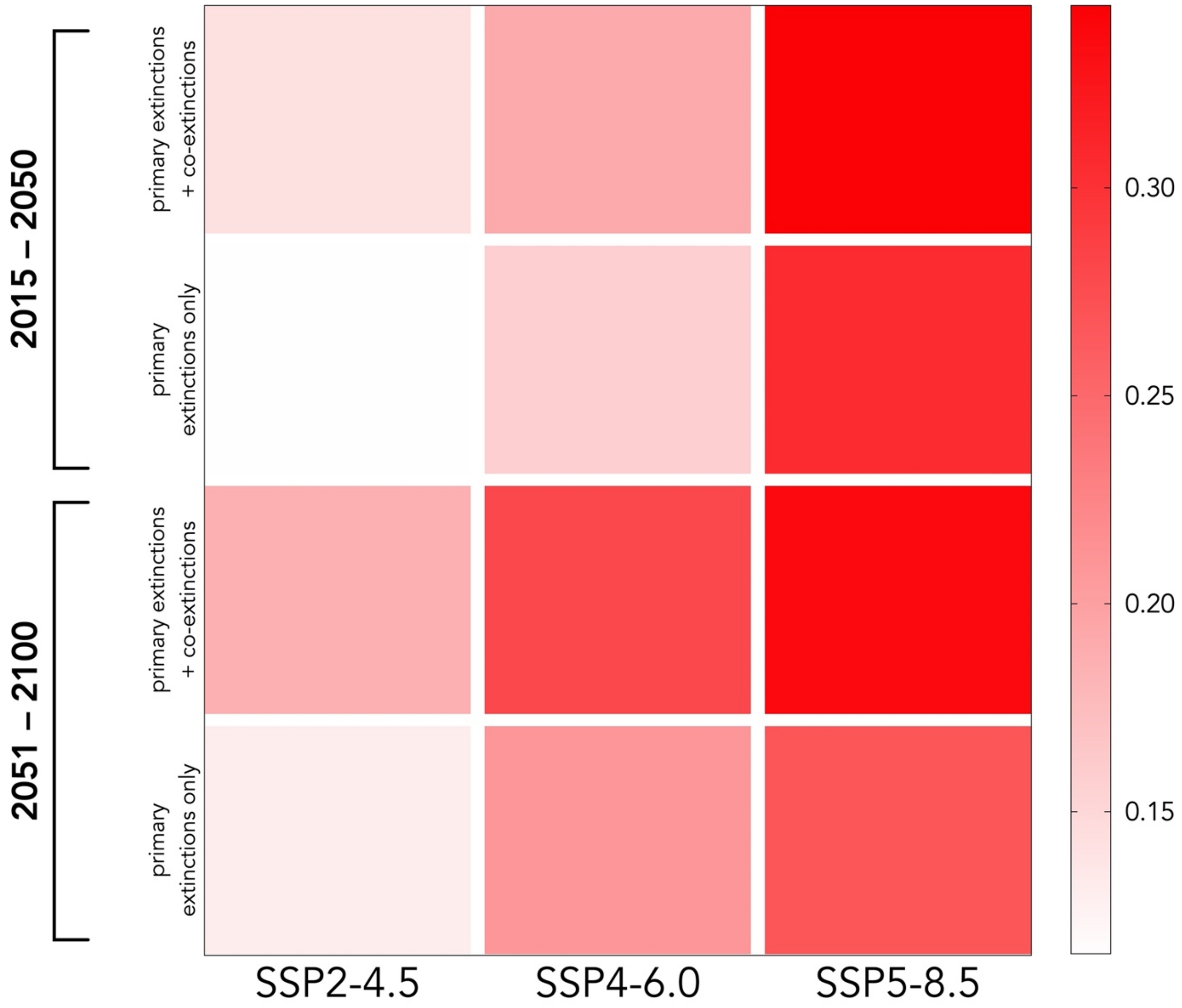

Fig. S12. Global average rate of vertebrate diversity loss before and after 2050. Compares rate of diversity loss between different future climate-change scenarios, either accounting or not for co-extinctions (2020–2050 and 2051–2100). These correspond to the slopes of the semi curves in Fig. 2 (bottom left panel) split by the 2050 boundary line.

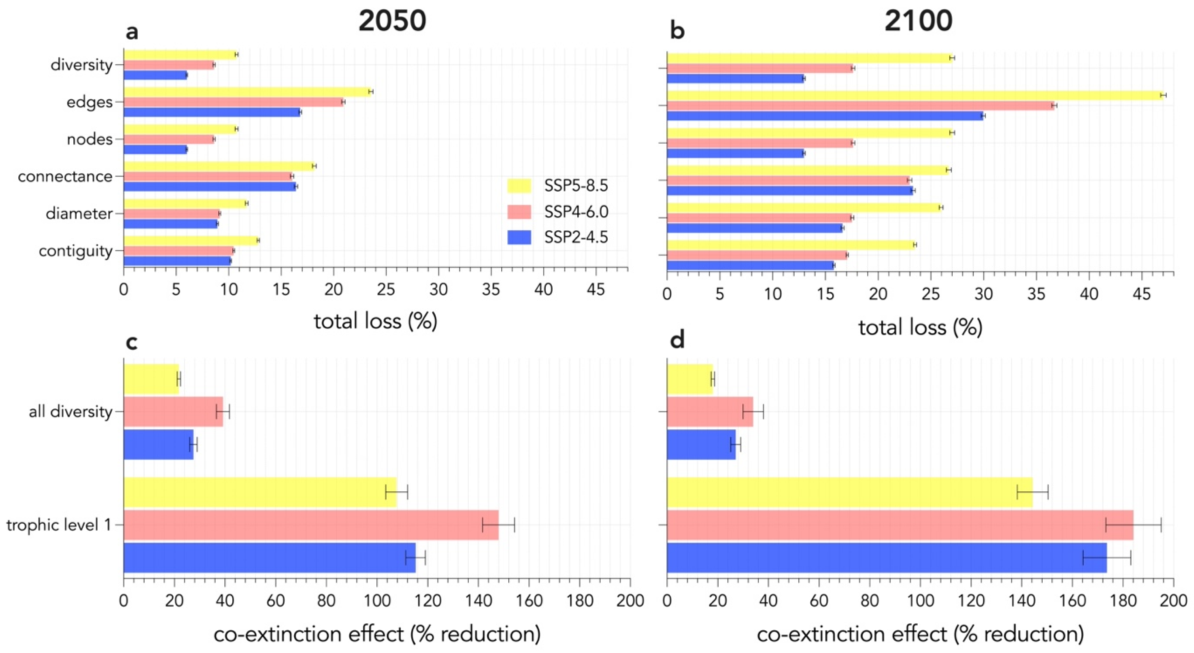

Fig. S13. Global-scale changes in vertebrate diversity and structure of vertebrate food webs until and after 2050. Relative reduction of various food-web properties: number of edges and nodes, connectance, diameter, fraction of nodes in the largest connected component (‘contiguity’; **a**, **b**), as well as the co-extinction effect (percentage increase in diversity loss in the co-extinction scenario compared to the reference scenario accounting only for primary extinctions; **c**, **d**) for the period 2020–2050 (**a**, **c**) compared to the period 2020–2100 (**b**, **d**). Whiskers indicate standard error.

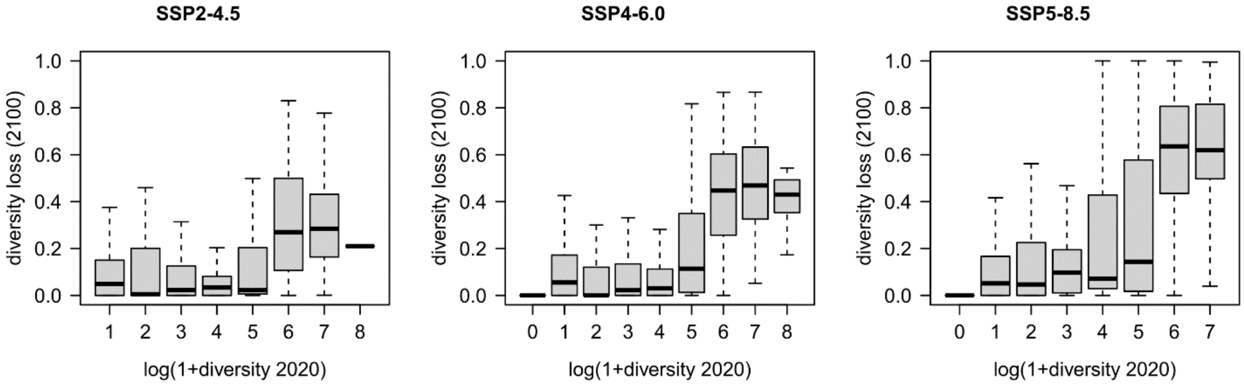

Fig. S14. Relative proportional diversity loss highest in species-rich localities. Expected diversity loss by 2100 for all localities worldwide (averaged across the 100 simulations per climatic scenario and relative to the 2020 reference local diversity) grouped according to their initial diversity (log_e-transformed). Boxes indicate first and third quartiles, whiskers indicate range, and horizontal lines indicate medians.

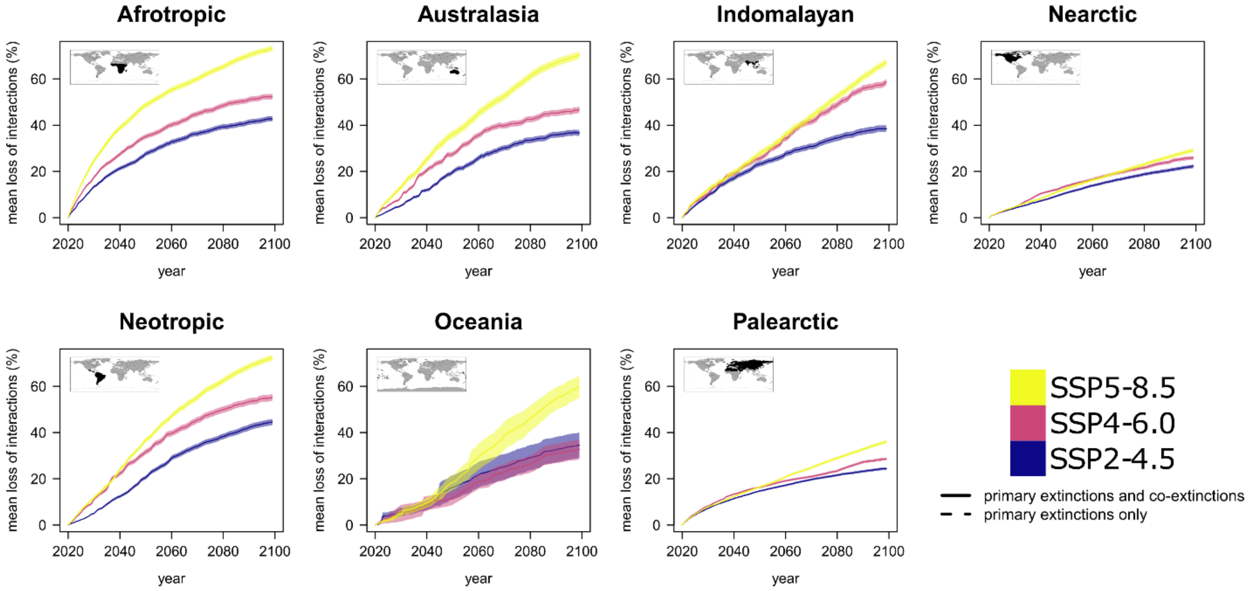

Fig. S15. Regional decline in ecological interactions. Loss refers to the reference local conditions in 2020. We first averaged local loss in the 100 replicates, and then averaged local loss values across all localities in a given region. Solid lines report averages, while shaded areas are 95% confidence intervals, showing the variation across localities within a region in the average map obtained with 100 simulations.

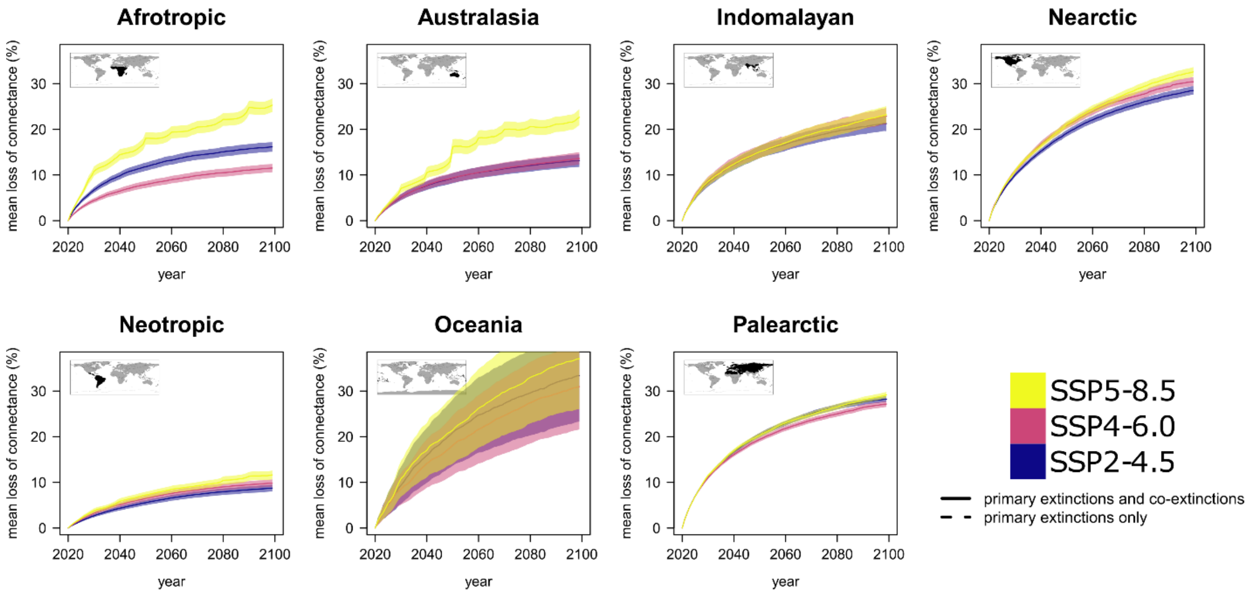

Fig. S16. Regional decline in connectance. Loss refers to the reference local conditions in 2020. We first averaged local loss in the 100 replicates, and then averaged local losses across all localities in a given region. Solid lines show averages, while shaded areas are 95% confidence intervals, showing the variation across localities within a region in the average map obtained with 100 simulations.

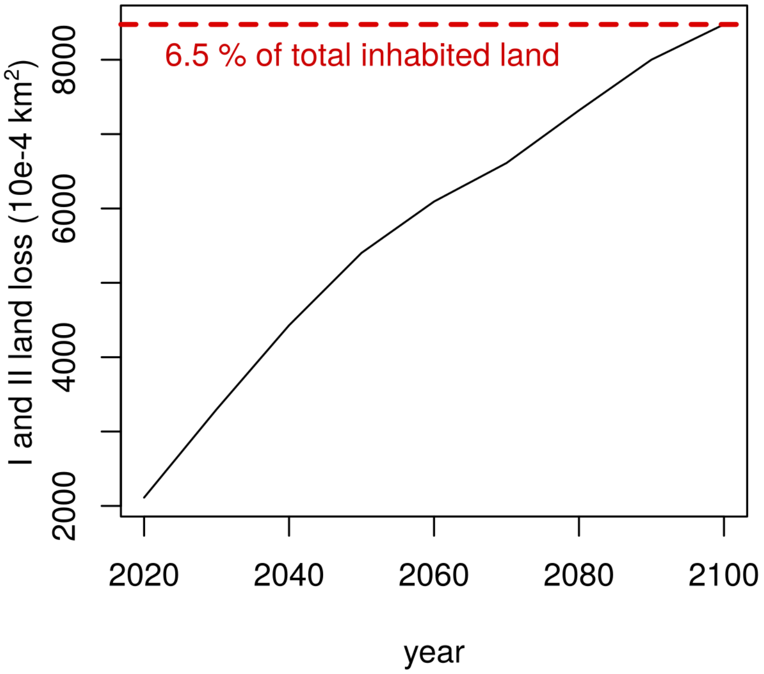

Fig. S17. Global extent of land-use change. Annual amount of modeled primary and secondary land lost from 2020 to the end of the century for the worst-case climate-change scenario (SSP5-8.5). Cumulative losses account for 8,000,000 km^2 in total; however, this value represents only 6.5% of the global area in the simulation inhabited by at least one species in 2020 (~ 130,000,000 km^2).

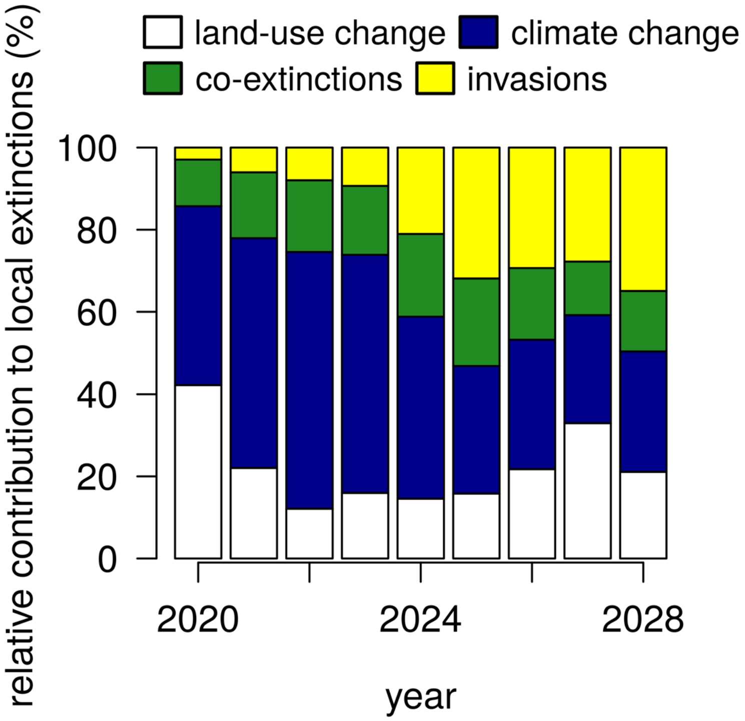

Fig. S18. Relative impact on global biodiversity loss of the different extinction drivers under an extreme assumption of the effect on land use change on local biodiversity. Barplots obtained from a single simulation with an instantaneous, complete biodiversity loss in a target locality following any loss of primary and secondary land under an SSP4-6.0 CMIP6 scenario.

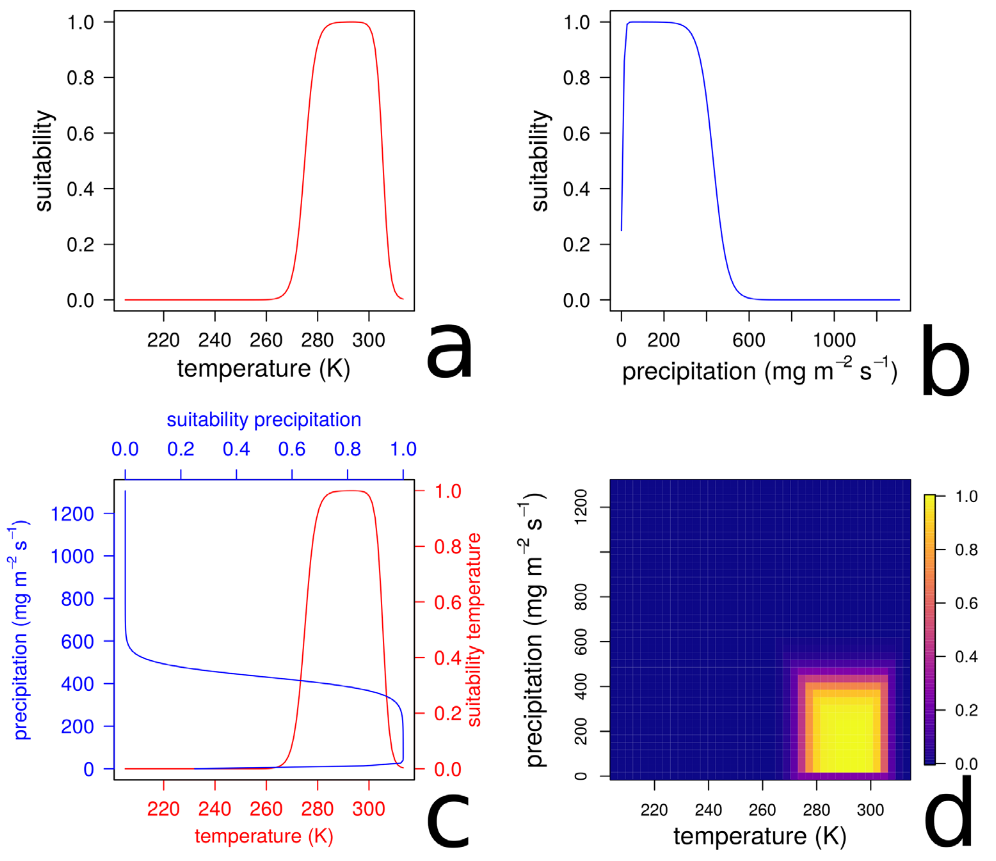

Fig. S19. Example of a virtual species' bidimensional climatic niche. The two curves (a, b) modeling the probability of survival of the target species in response to temperature and precipitation are drawn according to the procedure and equations described in the Methods. These are then combined (c) and, for each combination of temperature and precipitation, the probability of species survival is computed as the minimum value of the two curves (d).

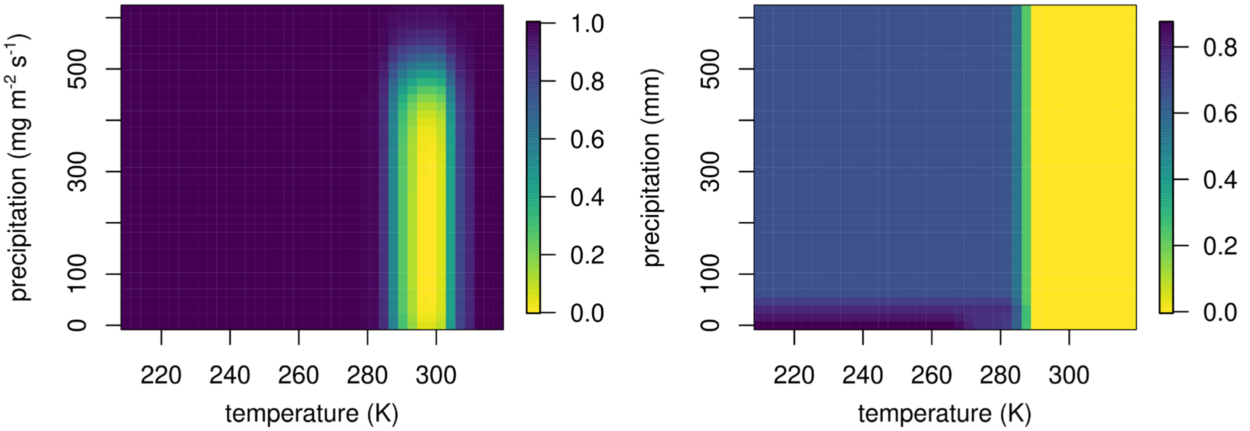

Fig. S20. Comparing alternative bidimensional climatic niches of a randomly selected virtual species. Left panel shows probability of survival of the target species in response to temperature and precipitation drawn according to the procedure and equations described in the Methods. Right panel shows probability of survival as modelled by a *RandomForest* regressor. Survival probability computed across a continuous range of temperature and precipitation (400 unique combinations of temperature and precipitation covering the ideal space from the minimum to the maximum values recorded on Earth in the reference period 2015–2019).

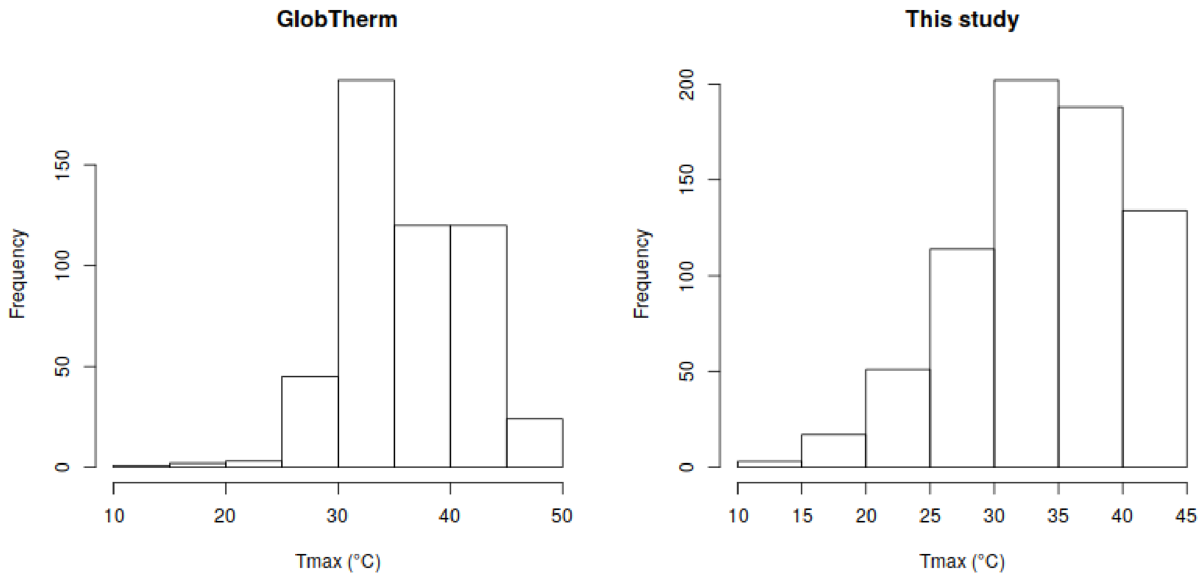

Fig. S21. Comparing distribution of species' thermal tolerances from the model *versus* empirical estimates. Left panel shows distribution of vertebrate thermal tolerances obtained from *GlobTherm* (nature.com/articles/sdata201822); right panel shows thermal tolerances obtained by combining IUCN species ranges with the CMIP6 climate projections (for the reference period 2015–2020; histogram refers to an intermediate scenario SSP4-6.0).

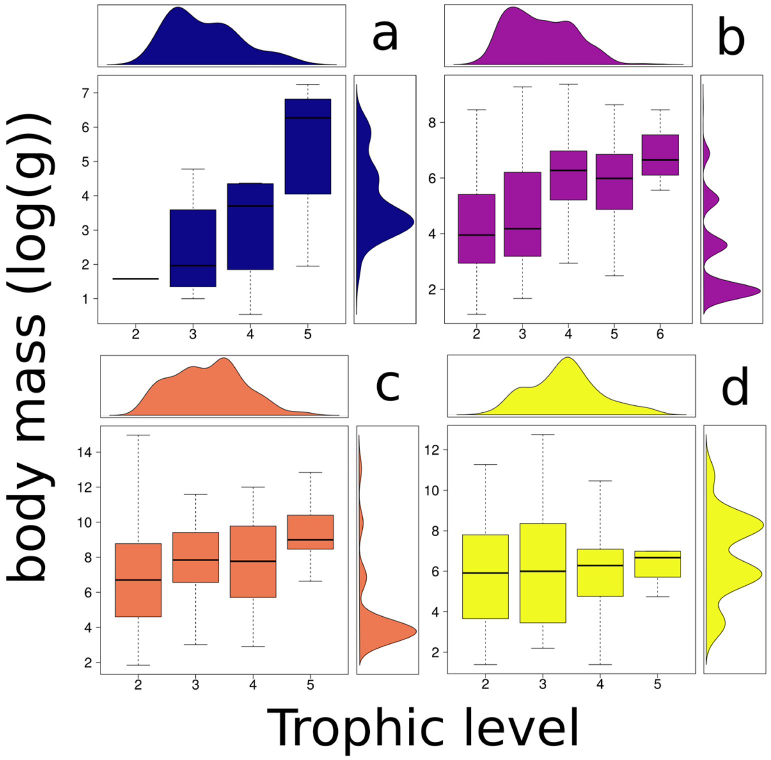

Fig. S22. Distribution of trophic levels associated with different body-size classes for different vertebrate taxa. Data include all vertebrate species we used to generate virtual species. (a) amphibians; (b) birds; (c) mammals; (d) reptiles. Boxes indicate first and third quartiles, whiskers indicate ranges, and horizontal lines indicate medians. See Methods for details on the sources for body size and trophic data.

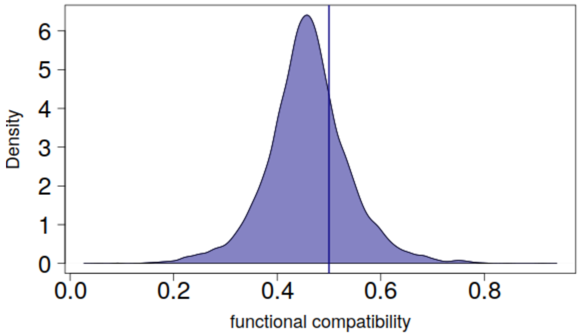

Fig. S23. Expected density of pairwise functional compatibility between any two virtual species. Distribution obtained by generating one million pairs of random species with randomly assigned phenotype, and computing functional compatibility as described in the Methods.

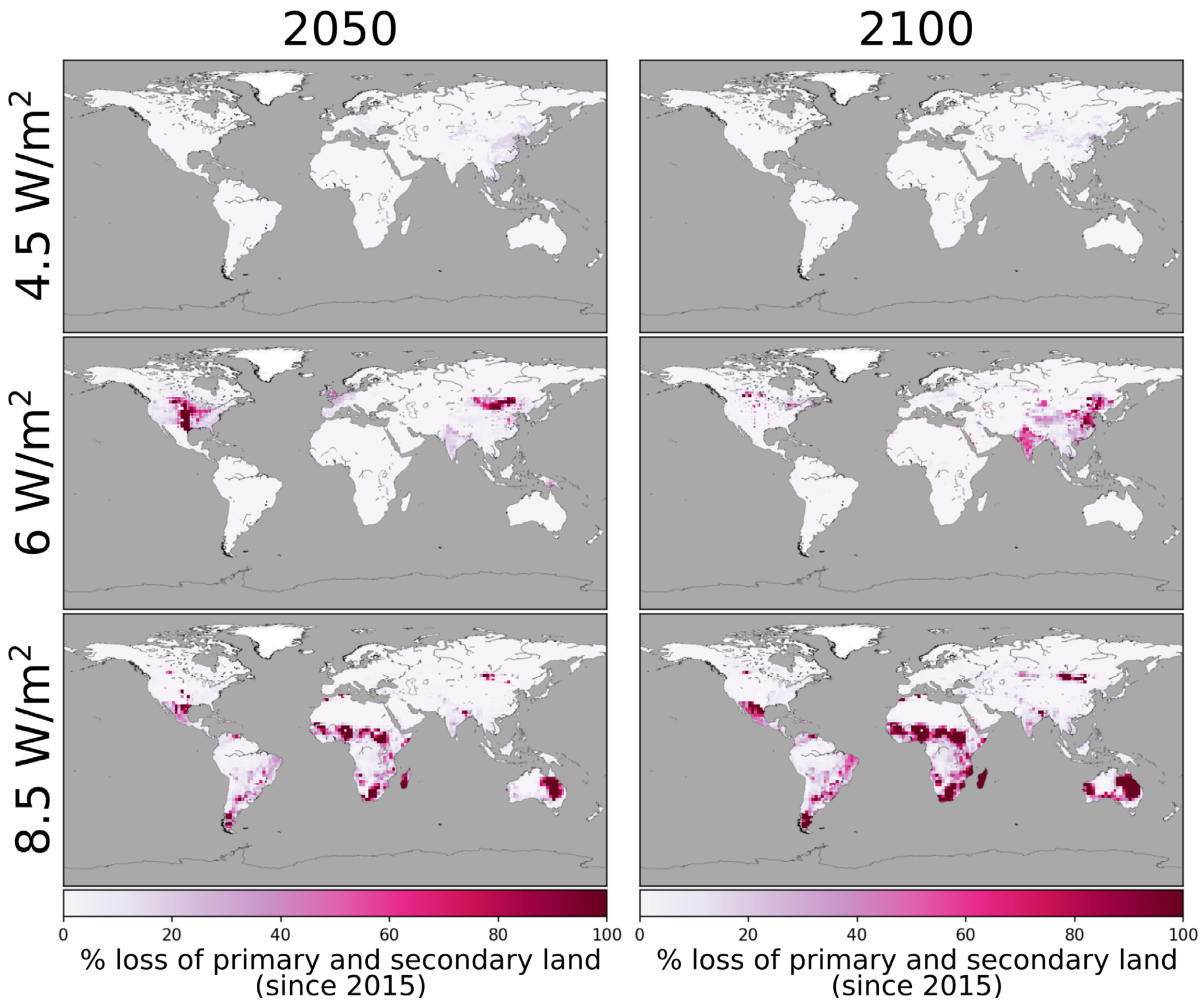

Fig. S24. Predicted land-use change by 2050 and 2100 under different emissions scenarios. Percentage loss of primary and secondary land (natural vegetation never impacted by humans, and natural vegetation recovering from previous anthropogenic disturbance, respectively) in 2050 and 2100 compared to 2015, for three Integrated Assessment Model scenarios with radiative forcing by year 2100 consistent with the SSP scenarios used for climate projections.

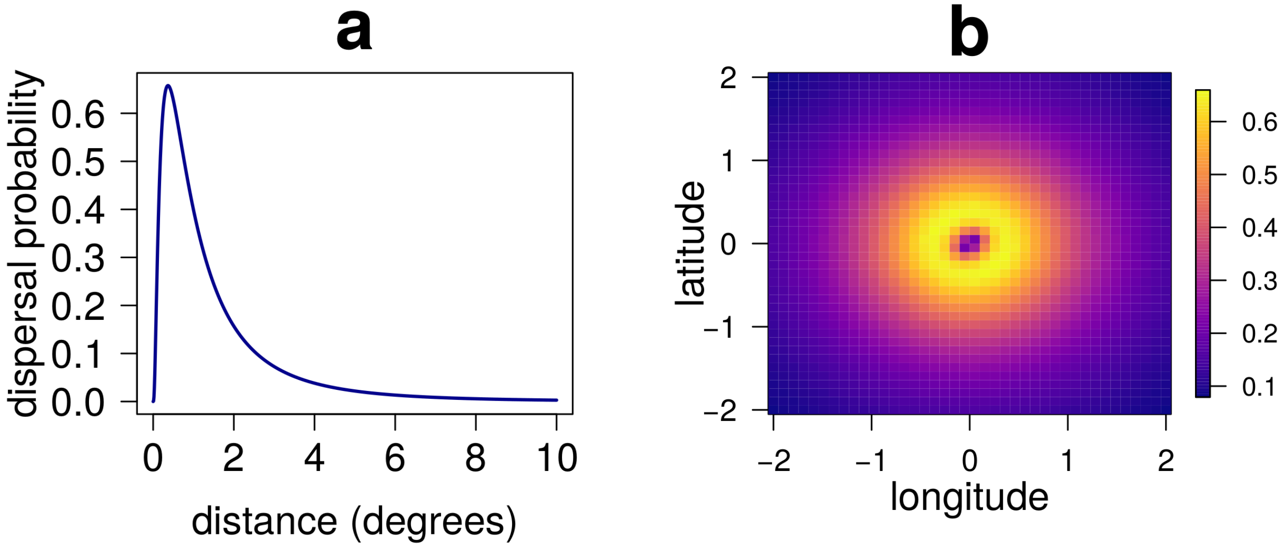

Fig. S25. Kernels for simulating dispersal and colonization patterns. Probability of a species moving from one location in the simulated Earth to another at a distance *d*+1 obtained by sampling *d* from a log-Normal distribution with mean = 0 and standard deviation = 1.

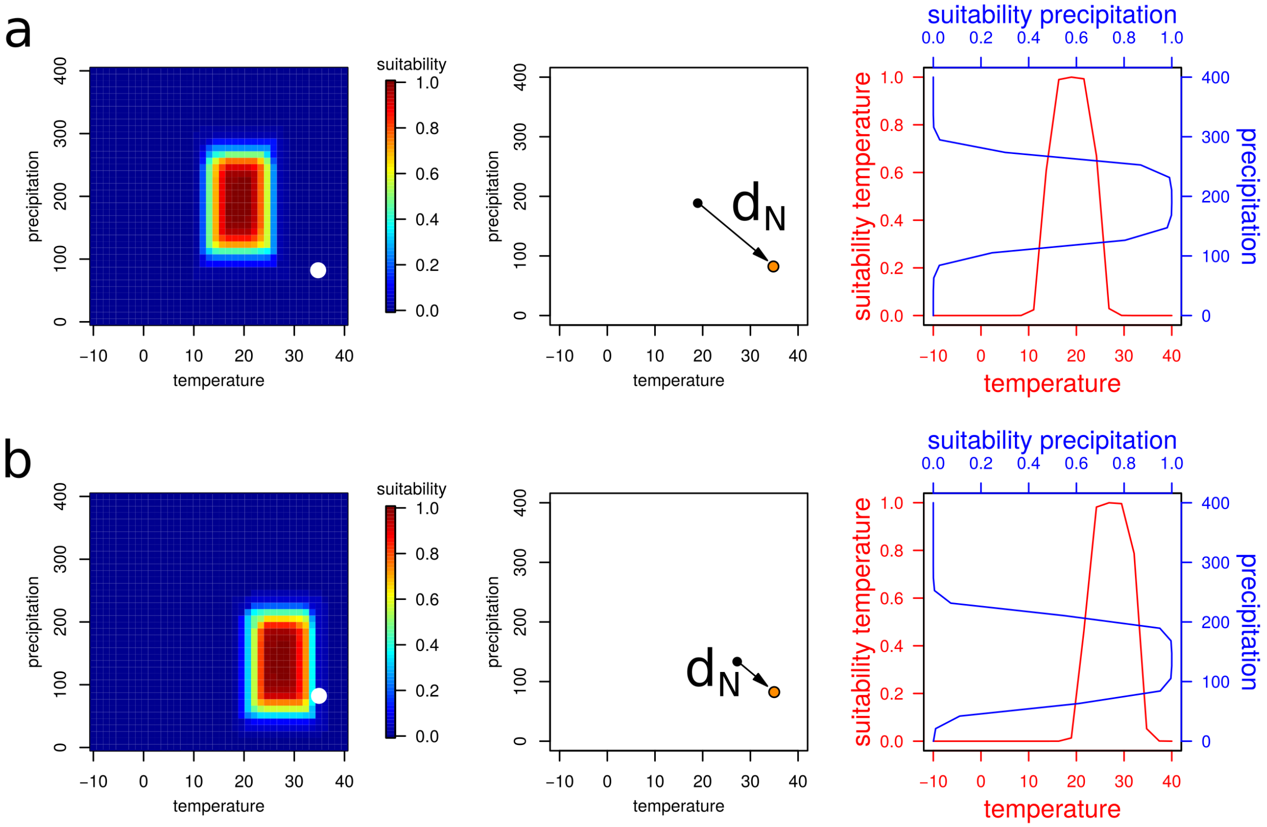

Fig. S26. Niche acclimation to local conditions. At each yearly step in the simulation, each virtual species in each locality shifts the center of its niche closer to the average local conditions recorded in the previous year with probability $p_{adp} = 0.001$. Both initial average and upper/lower tolerance limits for both temperature and precipitation (**a**) are shifted along the segment connecting the center of the species' niche and the mean local temperature and precipitation of the preceding year at a length given by $C_{adp} \times A \times d_N$, where C_{adp} = an overall simulation acclimation factor set to 0.01 (fixed for all simulations), A = species-specific adaptability (a random value between 0 and 1 assigned when creating the virtual species), and d_N = total distance between the center of the species' niche and mean local conditions. Finally, the niche parameters defining the curves of temperature and precipitation suitability are recomputed based on the updated parameters (**b**; see Methods).

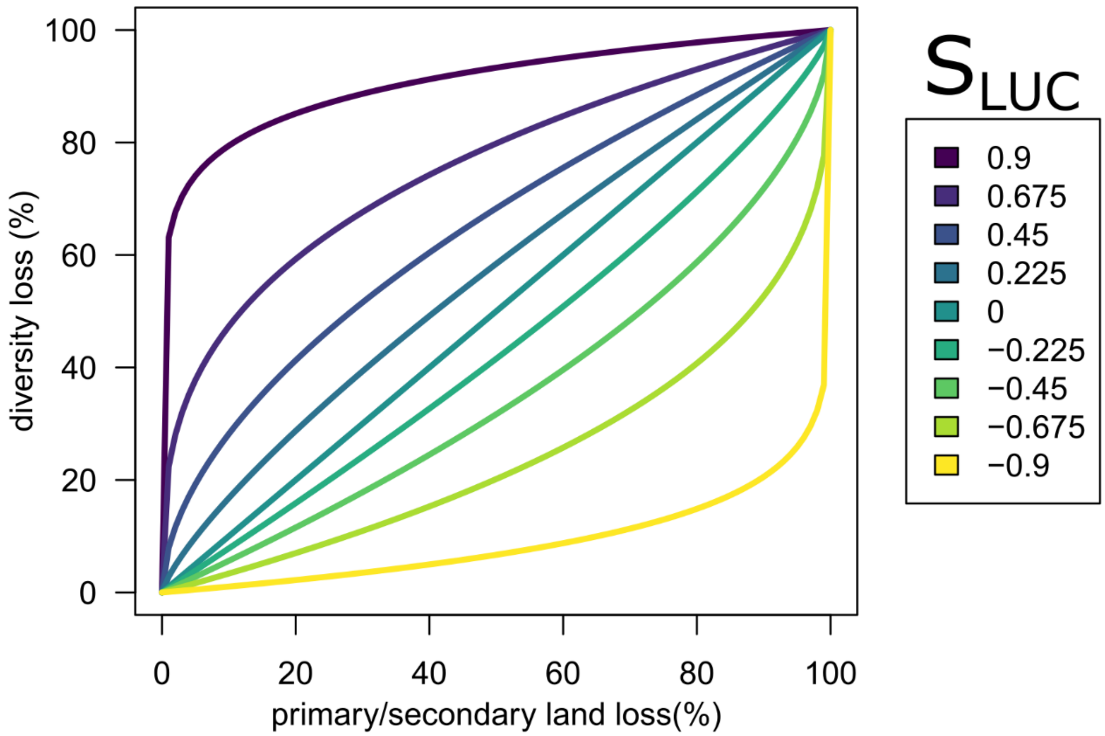

Fig. S27. Different response curves of local diversity to primary and secondary habitat loss. Curves have the form $loss = 1 - (1 - P_{LUC})^{R_1}$ if R_2 = -1, or $loss = P_{LUC}^{R_1}$ if R_2 = 1, where $loss$ = fraction of biodiversity lost following habitat loss via land-use change; P_{LUC} = fraction of primary and secondary land lost at a given time step compared to the previous one; R_1 = a random number in the range {0,1}; and R_2 sampled from {-1,1} with equal probability. Parameters R_1 and R_2 are combined into a single parameter $S_{LUC} = R_2(1 - R_1)$ describing the response curves of diversity loss continuously from 1 (full diversity loss occurs even with no habitat loss) to -1 (diversity loss is invariant to full habitat loss), with S_{LUC} = 0 representing the linear response used in the main simulations.

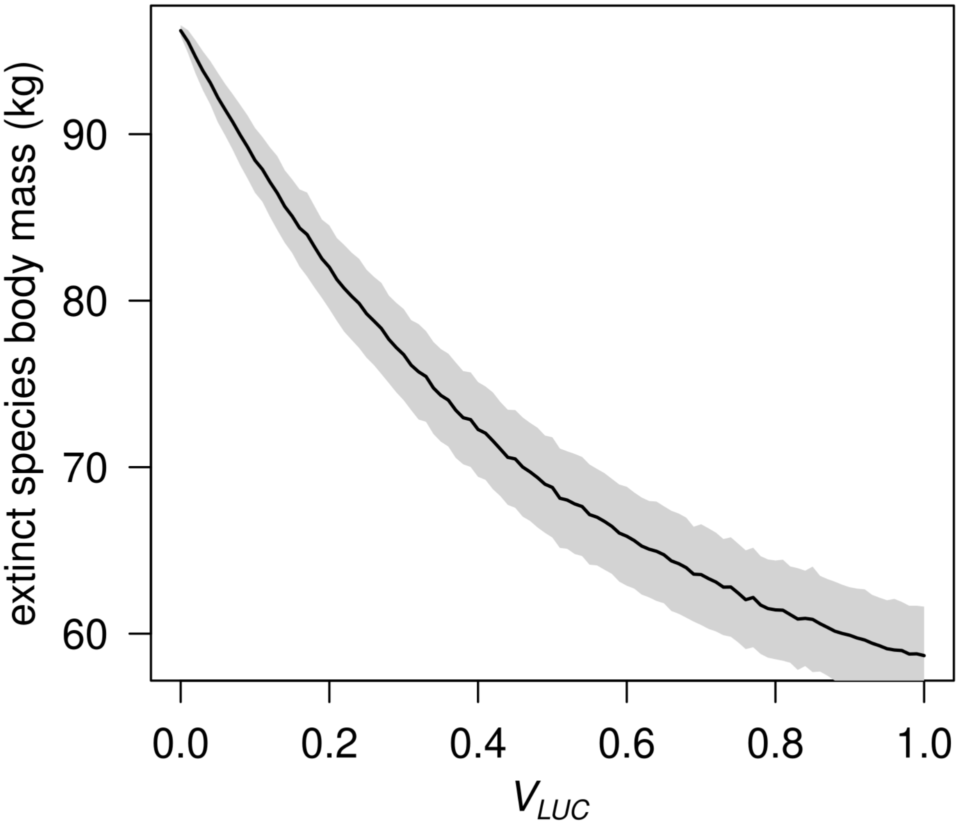

Fig. S28. Example of how V_{LUC} parameter modulates the relationship between vertebrate body mass and vulnerability to land-use change. Shows average body size of 100 species going extinct due to land-use change in a locality hosting a hypothetical set of 1000 species with body size randomly varying between 1 and 100 kg for increasing V_{LUC} (see Methods). Continuous line is the average of 1000 replicates, while the shaded areas represent standard deviation.

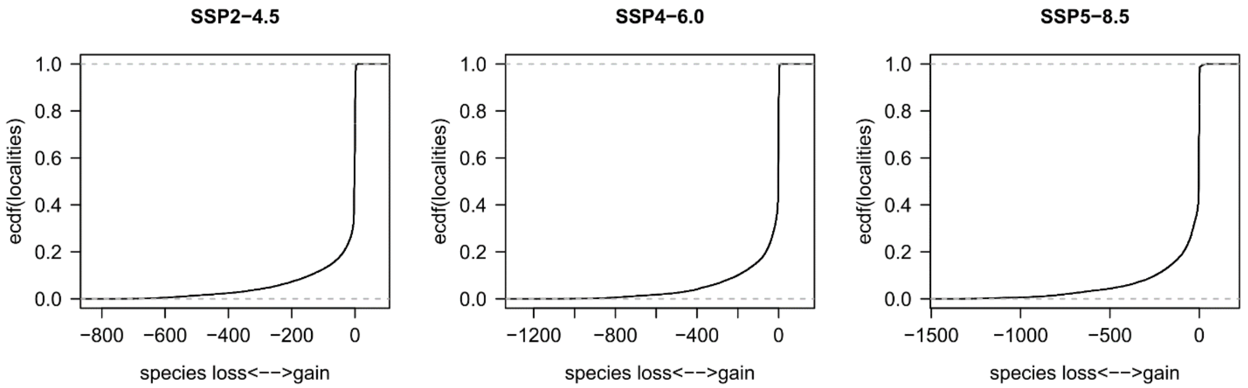

Fig. S29. Future losses will largely outweigh gains in diversity. Shows empirical cumulative distribution function curves indicating that most localities will experience loss of diversity and not gain, and that gains will be limited to a few species per locality.

Table S1. Relative contribution of different extinction drivers to species loss. Shows relative yearly percentage of total recorded local extinction events (averaged across 100 simulations) for each extinction driver. Invasions refer to the replacement of a local species by a colonizer from a different locality.

	**land-use change**		**climate change**		**co-extinctions**		**invasions**	
**scenario**	**mean**	**SD**	**mean**	**SD**	**mean**	**SD**	**mean**	**SD**
SSP2-4.5	0.4	0.6	57.8	8.2	22.8	7.7	19.1	3.7
SSP4-6.0	3.7	3.1	62.1	7.2	20.3	5.4	13.9	4.1
SSP5-8.5	11.6	7.3	56.7	11.5	19.8	8.2	11.9	2.3

REFERENCES AND NOTES

1. A. D. Barnosky, N. Matzke, S. Tomiya, G. O. U. Wogan, B. Swartz, T. B. Quental, C. Marshall, J. L. McGuire, E. L. Lindsey, K. C. Maguire, B. Mersey, E. A. Ferrer, Has the Earth's sixth mass extinction already arrived?. *Nature* **471**, 51–57 (2011).

2. G. Ceballos, P. R. Ehrlich, R. Dirzo, Biological annihilation via the ongoing sixth mass extinction signaled by vertebrate population losses and declines. *Proc. Natl. Acad. Sci. U.S.A.* **114**, E6089–E6096 (2017).

3. S. Díaz, J. Settele, E. S. Brondízio, H. T. Ngo, J. Agard, A. Arneth, P. Balvanera, K. A. Brauman, S. H. M. Butchart, K. M. A. Chan, L. A. Garibaldi, K. Ichii, J. Liu, S. M. Subramanian, G. F. Midgley, P. Miloslavich, Z. Molnár, D. Obura, A. Pfaff, S. Polasky, A. Purvis, J. Razzaque, B. Reyers, R. Roy Chowdhury, Y-J Shin, I. Visseren-Hamakers, K. J. Willis, C. N. Zayas, Pervasive human-driven decline of life on Earth points to the need for transformative change. *Science* **366**, eaax3100 (2019).

4. G. Ceballos, P. R. Ehrlich, P. H. Raven, Vertebrates on the brink as indicators of biological annihilation and the sixth mass extinction. *Proc. Natl. Acad. Sci. U.S.A.* **117,** 13596–13602 (2020).

5. G. Strona, *Hidden Pathways to Extinction* (Springer, 2022), pp. 237.

6. N. S. Sodhi, B. W. Brook, C. J. A. Bradshaw, Causes and consequences of species extinctions, in *The Princeton Guide to Ecology*, S. A. Levin, Ed. (Princeton Univ. Press, 2009), pp. 514–520.

7. B. W. Brook, N. S. Sodhi, C. J. A. Bradshaw, Synergies among extinction drivers under global change. *Trends Ecol. Evol.* **25**, 453–460 (2008).

8. R. R. Dunn, N. C. Harris, R. K. Colwell, L. P. Koh, N. S. Sodhi, The sixth mass coextinction: Are most endangered species parasites and mutualists?. *Proc. Roy. Soc. B* **276**, 3037–3045 (2009).

9. R. K. Colwell, R. R. Dunn, N. C. Harris, Coextinction and persistence of dependent species in a changing world. *Ann. Rev. Ecol. Evol. Syst.* **43**, 183–203 (2012).

10. J. F. Brodie, C. E. Aslan, H. S. Rogers, K. H. Redford, J. L. Maron, J. L. Bronstein, C. R. Groves, Secondary extinctions of biodiversity. *Trends Ecol. Evol.* **29**, 664–672 (2014).

11. G. Strona, C. J. Bradshaw, Co-extinctions annihilate planetary life during extreme environmental change. *Sci. Rep.* **8**, 1–12 (2018).

12. C. D. Thomas, A. Cameron, R. E. Green, M. Bakkenes, L. J. Beaumont, Y. C. Collingham, B. F. N. Erasmus, M. F. de Siqueira, A. Grainger, L. Hannah, L. Hughes, B. Huntley, A. S. van Jaarsveld, G. F. Midgley, L. Miles, M. A. Ortega-Huerta, A. Townsend Peterson, O. L. Phillips, S. E. Williams, Extinction risk from climate change. *Nature* **427**, 145–148 (2004).

13. J. R. Malcolm, C. Liu, R. P. Neilson, L. Hansen, L. Hannah, Global warming and extinctions of endemic species from biodiversity hotspots. *Conserv. Biol.* **20**, 538–548 (2006).

14. D. P. van Vuuren, O. E. Sala, H. M. Pereira, The future of vascular plant diversity under four global scenarios. *Ecol. Soc.* **11**, 25 (2006).

15. W. Jetz, D. S. Wilcove, A. P. Dobson, Projected impacts of climate and land-use change on the global diversity of birds. *PLoS Biol.* **5**, e157 (2007).

16. B. Sinervo, F. Méndez-de-la-Cruz, D. B. Miles, B. Heulin, E. Bastiaans, M. Villagrán-Santa Cruz, R. Lara-Resendiz, N. Martínez-Méndez, M. L. Calderón-Espinosa, R. N. Meza-Lázaro, H. Gadsden, L. J. Avila, M. Morando, I. J. De la Riva, P. Victoriano Sepulveda, C. F. Rocha, N. Ibargüengoytía, C. Aguilar Puntriano, M. Massot, V. Lepetz, T. A. Oksanen, D. G. Chapple, A. M. Bauer, W. R. Branch, J. Clobert, J. W. Sites Jr., Erosion of lizard diversity by climate change and altered thermal niches. *Science* **328**, 894–899 (2010).

17. A. Jarvis, A. Lane, R. J. Hijmans, The effect of climate change on crop wild relatives. *Agric. Ecosyst. Environ.* **126**, 13–23 (2008).

18. F. Ihlow, J. Dambach, J. O. Engler, M. Flecks, T. Hartmann, S. Nekum, H. Rajaei, D. Rödder, On the brink of extinction? How climate change may affect global chelonian species richness and distribution. *Glob. Change Biol.* **18**, 1520–1530 (2012).

19. W. B. Foden, S. H. Butchart, S. N. Stuart, J. C. Vié, H. R. Akçakaya, A. Angulo, L. M. DeVantier, A. Gutsche, E. Turak, L. Cao, S. D. Donner, V. Katariya, R. Bernard, R. A. Holland, A. F. Hughes, S. E. O'Hanlon, S. T. Garnett, C. H. Sekercioğlu, G. M. Mace, Identifying the world's most climate

change vulnerable species: A systematic trait-based assessment of all birds, amphibians and corals. *PLOS ONE* **8**, e65427 (2013).

20. R. Warren, J. VanDerWal, J. Price, J. A. Welbergen, I. Atkinson, J. Ramirez-Villegas, T. J. Osborn, A. Jarvis, L. P. Shoo, S. E. Williams, J. Lowe, Quantifying the benefit of early climate change mitigation in avoiding biodiversity loss. *Nat. Clim. Change* **3**, 678–682 (2013).

21. M. C. Urban, Accelerating extinction risk from climate change. *Science* **348**, 571–573 (2015).

22. D. Purves, J. P. W. Scharlemann, M. Harfoot, T. Newbold, D. P. Tittensor, J. Hutton, S. Emmott, Time to model all life on Earth. *Nature* **493**, 295–297 (2013).

23. G. Strona, P. S. A. Beck, M. Cabeza, S. Fattorini, F. Guilhaumon, F. Micheli, S. Montano, O. Ovaskainen, S. Planes, J. A. Veech, V. Parravicini, Ecological dependencies make remote reef fish communities most vulnerable to coral loss. *Nat. Commun.* **12**, 1–10 (2021).

24. J. Llewelyn, G. Strona, M. C. McDowell, C. N. Johnson, K. J. Peters, D. B. Stouffer, S. N. de Visser, F. Saltré, C. J. A. Bradshaw, Sahul's megafauna were vulnerable to plant-community changes due to their position in the trophic network. *Ecography* **2022**, e06089 (2022).

25. V. Eyring, S. Bony, G. A. Meehl, C. A. Senior, B. Stevens, R. J. Stouffer, K. E. Taylor, Overview of the coupled model intercomparison Project Phase 6 (CMIP6) experimental design and organization. *Geosci. Model Dev.* ***9***, 1937–1958 (2016).

26. R. van Klink, D. E. Bowler, K. B. Gongalsky, A. B. Swengel, A. Gentile, J. M. Chase, Meta-analysis reveals declines in terrestrial but increases in freshwater insect abundances. *Science* **368**, 417–420 (2020).

27. N. Myers, The biodiversity challenge: Expanded hot-spots analysis. *Environmentalist* **10**, 243–256 (1990).

28. X. Giam, C. J. Bradshaw, H. T. Tan, N. S. Sodhi, Future habitat loss and the conservation of plant biodiversity. *Biol. Conserv.* **143**, 1594–1602 (2010).

29. C. Bellard, P. Cassey, T. M. Blackburn, Alien species as a driver of recent extinctions. *Biol. Lett.* **12**, 20150623 (2016).

30. S. Kumschick, M. Gaertner, M. Vilà, F. Essl, J. M. Jeschke, P. Pyšek, A. Ricciardi, S. Bacher, T. M. Blackburn, J. T. Dick, T. Evans, Ecological impacts of alien species: Quantification, scope, caveats, and recommendations. *Bioscience* **65**, 55–63 (2015).

31. G. Strona, K. D. Lafferty, Environmental change makes robust ecological networks fragile. *Nat. Commun.* **7**, 1–7 (2016).

32. IUCN. The IUCN Red List of Threatened Species. Version 2020–3. www.iucnredlist.org. Downloaded on 01/05/2020 (2020).

33. BirdLife International and Handbook of the Birds of the World. Bird species distribution maps of the world. Version 2018.1. http://datazone.birdlife.org/species/requestdis (2018).

34. Canadian Centre for Climate Modelling and Analysis. CMIP6 climate scenarios. https://climate-scenarios.canada.ca/?page=cmip6-scenarios. Downloaded on 07/09/2022 (2022).

35. M. B. Araújo, A. T. Peterson, Uses and misuses of bioclimatic envelope modeling. *Ecology* **93**, 1527–1539 (2012).

36. A. Guisan, W. Thuiller, Predicting species distribution: Offering more than simple habitat models. *Ecol. Lett.* **8**, 993–1009 (2005).

37. J. M. Bennett, P. Calosi, S. Clusella-Trullas, B. Martínez, J. Sunday, A. C. Algar, M. B. Araújo, B. A. Hawkins, S. Keith, I. Kühn, C. Rahbek, GlobTherm, a global database on thermal tolerances for aquatic and terrestrial organisms. *Sci. Data* **5**, 1–7 (2018).

38. K. E. Jones, J. Bielby, M. Cardillo, S. A. Fritz, J. O'Dell, C. D. L. Orme, K. Safi, W. Sechrest, E. H. Boakes, C. Carbone, C. Connolly, M. J. Cutts, J. K. Foster, R. Grenyer, M. Habib, C. A. Plaster, S. A. Price, E. A. Rigby, J. Rist, A. Teacher, O. R. P. Bininda-Emonds, J. L. Gittleman, G. M. Mace, A. Purvis, PanTHERIA: A species- level database of life history, ecology, and geography of extant and recently extinct mammals. *Ecology* **90**, 2648–2648 (2009).

39. H. Wilman, J. Belmaker, J. Simpson, C. de la Rosa, M. M. Rivadeneira, W. Jetz, EltonTraits 1.0: Species-level foraging attributes of the world's birds and mammals. *Ecology* **95**, 2027–2027 (2014).

40. A. Slavenko, O. J. Tallowin, Y. Itescu, P. Raia, S. Meiri, Late quaternary reptile extinctions: Size matters, insularity dominates. *Global Ecol. Biogeog.* **25**, 1308–1320 (2016).

41. B. F. Oliveira, V. A. São-Pedro, G. Santos-Barrera, C. Penone, G. C. Costa, AmphiBIO, a global database for amphibian ecological traits. *Sci. Data* **4**, 170123 (2017).

42. J. H. Poelen, J. D. Simons, C. J. Mungall, Global biotic interactions: An open infrastructure to share and analyze species-interaction datasets. *Ecol. Inform.* **24**, 148–159 (2014).

43. R. M. May, Will a large complex system be stable? *Nature* **238**, 413–414 (1972).

44. L. P. Chini, G. C. Hurtt, S. Frolking, Harmonized Global Land Use for Years 1500–2100, V1. Data set. Available online [http://daac.ornl.gov] from Oak Ridge National Laboratory Distributed Active Archive Center, Oak Ridge, Tennessee, USA (2014).

45. M. Kuussaari, R. Bommarco, R. K. Heikkinen. A. Helm, J. Krauss, R. Lindborg, E. Öckinger, M. Pärtel, J. Pino, F. Rodà, C. Stefanescu, T. Teder, M. Zobel, I. Steffan-Dewenter, Extinction debt: A challenge for biodiversity conservation. *Trends Ecol. Evol.* **24**, 564–571 (2009).

46. C. J. A. Bradshaw, P. R. Ehrlich, A. Beattie, G. Ceballos, E. Crist, J. Diamond, R. Dirzo, A. H. Ehrlich, J. Harte, M. E. Harte, G. Pyke, Underestimating the challenges of avoiding a ghastly future. *Front. Conserv. Sci.* **1**, 615419 (2021).

47. J. Elith, J. R. Leathwick, T. Hastie, A working guide to boosted regression trees. *J. Anim. Ecol.* **77**, 802–813 (2008).

48. R. J. Hijmans, S. Phillips, J. Leathwick, J. Elith, dismo: Species distribution modeling. R package version 0.9–3. http://CRAN.R-project.org/package=dismo (2013).

49. T. A. Prowse, C. J. A. Bradshaw, S. Delean, P. Cassey, R. C. Lacy, K. Wells, M. E. Aiello-Lammens, H. R. Akçakaya, B. W. Brook, An efficient protocol for the global sensitivity analysis of stochastic ecological models. *Ecosphere* **7**, e01238 (2016).

50. I. Morales-Castilla, M. G. Matias, D. Gravel, M. B. Araújo, Inferring biotic interactions from proxies. *Trends Ecol. Evol.* **30**, 347–356 (2015).

51. M. Dornelas, N. J. Gotelli, B. McGill, H. Shimadzu, F. Moyes, C. Sievers, A. E. Magurran, Assemblage time series reveal biodiversity change but not systematic loss. *Science* **344**, 296–299 (2014).

52. M. Dornelas, N. J. Gotelli, H. Shimadzu, F. Moyes, A. E. Magurran, B. J. McGill, A balance of winners and losers in the Anthropocene. *Ecol. Lett.* **22**, 847–854 (2019).
